# The Biogeochemical Sulfur Cycle of Marine Sediments

**DOI:** 10.3389/fmicb.2019.00849

**Published:** 2019-04-24

**Authors:** Bo Barker Jørgensen, Alyssa J. Findlay, André Pellerin

**Affiliations:** Department of Bioscience, Center for Geomicrobiology, Aarhus University, Aarhus, Denmark

**Keywords:** sulfate reduction, sulfide oxidation, sulfur disproportionation, sulfate reducing bacteria, sulfide oxidizing bacteria, stable isotopes, sulfur isotope fractionation

## Abstract

Microbial dissimilatory sulfate reduction to sulfide is a predominant terminal pathway of organic matter mineralization in the anoxic seabed. Chemical or microbial oxidation of the produced sulfide establishes a complex network of pathways in the sulfur cycle, leading to intermediate sulfur species and partly back to sulfate. The intermediates include elemental sulfur, polysulfides, thiosulfate, and sulfite, which are all substrates for further microbial oxidation, reduction or disproportionation. New microbiological discoveries, such as long-distance electron transfer through sulfide oxidizing cable bacteria, add to the complexity. Isotope exchange reactions play an important role for the stable isotope geochemistry and for the experimental study of sulfur transformations using radiotracers. Microbially catalyzed processes are partly reversible whereby the back-reaction affects our interpretation of radiotracer experiments and provides a mechanism for isotope fractionation. We here review the progress and current status in our understanding of the sulfur cycle in the seabed with respect to its microbial ecology, biogeochemistry, and isotope geochemistry.

## Introduction

The sulfur cycle of marine sediments is primarily driven by the dissimilatory sulfate reduction (DSR) to sulfide by anaerobic microorganisms (e.g., Jørgensen and Kasten, 2006). This process links the complex food web of organic matter degradation to the terminal organic carbon oxidation to CO_2_. Most of the sulfide is ultimately reoxidized back to sulfate, via diverse sulfur intermediates, by geochemical or microbial reactions that involve oxygen, nitrate, manganese [Mn(IV)], iron [Fe(III)], and other potential oxidants (e.g., Rickard, 2012). A fraction of the sulfide precipitates with iron and other metals or reacts with organic matter and is buried deeply into the seabed. The microbial sulfur transformations affect the isotopic composition of sulfate and sulfides and the resulting isotope fractionation is thereby diagnostic for both process rates and pathways of the sulfur cycle (e.g., Canfield, 2001).

We here review recent progress and selected aspects of these processes with emphasis on the interactions between microbial communities and the ambient sediment geochemistry. The processes are discussed with respect to their rates and pathways. We focus on fine-grained continental shelf sediments and do not discuss advective ecosystems such as cold seeps or hot springs or the low-energy ecosystems of the deep sea. Most examples are taken from coastal marine sediments of the Baltic Sea region. The cited data thereby provide a consistent picture of how the sulfur cycle may function in a specific seabed. With respect to the diversity and physiology of the respective microorganisms we refer to recent reviews (e.g., Finster, 2008; Muyzer and Stams, 2008; Knittel and Boetius, 2009; Rabus et al., 2015; Wasmund et al., 2017). More comprehensive overviews of the biogeochemical sulfur cycle in marine sediments have been published by, e.g., Canfield (2001), Amend et al. (2004), Canfield et al. (2005), and Jørgensen and Kasten (2006).

**FIGURE 1:**
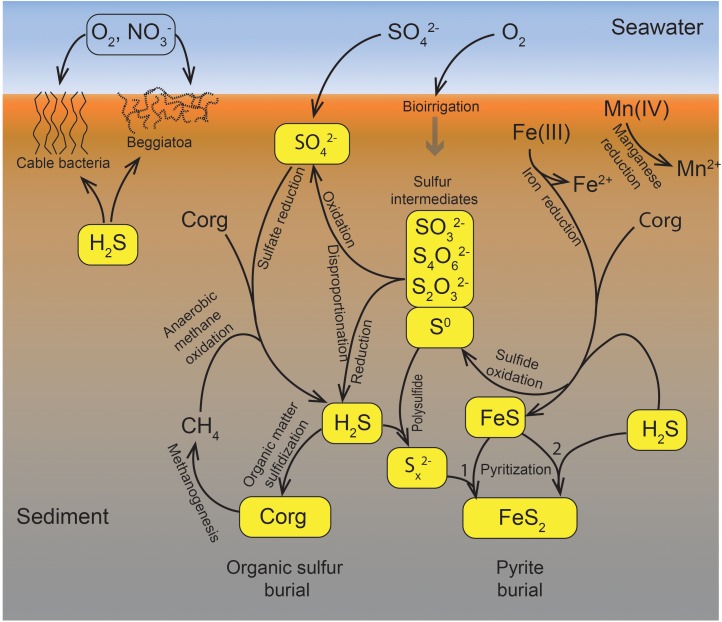
The biogeochemical sulfur cycle of marine sediments. The schematic presentation includes many of the processes discussed in this review. Arrows indicate fluxes and pathways of biological or chemical processes. For further explanation, see text.

Figure 1 presents the sulfur cycle of marine sediments, as it will be discussed in this review. The processes include chemical reactions, microbially catalyzed pathways, and a combination of both. Sulfate (SO_4_^2-^) reduction to sulfide (H_2_S + HS^-^ + S^2-^) is driven by the oxidation of buried organic carbon (C_org_), supplemented by the anaerobic oxidation of methane (CH_4_) at the subsurface sulfate-methane transition (SMT). Manganese and iron reduction are focused toward the surface sediment, but Fe(III) is also buried and acts as an oxidant for sulfide in the deeper sediment layers where it partly binds the produced sulfide as iron sulfide (FeS) and pyrite (FeS_2_). Pyrite is the end product of iron-sulfide mineral formation and provides a deep sink for sulfur. Two pathways of pyrite formation are discussed here, the “polysulfide pathway” (1) and the “H_2_S pathway” (2) (Figure 1). The sulfidization of buried organic matter provides an additional deep sink for sulfur. Intermediate sulfur species, such as elemental sulfur (S^0^), thiosulfate (S_2_O_3_^2-^), tetrathionate (S_4_O_6_^2-^), and sulfite (SO_3_^2-^), are formed during the oxidation of sulfide by, for example, buried Fe(III). These intermediates may be reduced back to sulfide, oxidized further to sulfate, or disproportionated to form both sulfide and sulfate. In very sulfidic sediments, a part of the sulfide diffuses up to the surface sediment where it may be oxidized by cable bacteria, by large sulfur bacteria such as *Beggiatoa* spp., or by other, less conspicuous sulfide oxidizers. The different pathways of sulfide oxidation ultimately depend on oxygen (and less on nitrate) as the ultimate oxidant, and thereby consume a considerable part of the total oxygen uptake of the seabed (Jørgensen, 1982b). The oxygen flux into the sediment is enhanced by bioirrigation (ventillation of burrows) by the benthic macrofauna (e.g., Kristensen et al., 2013).

## Sulfate Reduction

### Organic Matter Degradation

Organic matter deposited on the seafloor provides food for the benthic communities, either at the sediment surface or upon burial into the sediment layers below. Oxygen is available for respiration and chemical reactions near the surface and through faunal burrows. Beneath this mixed surface zone, marine sediments constitute an anoxic world inhabited by anaerobic microorganisms. These subsurface organisms become increasingly sparse with depth, yet they account for half of all microbial cells in the ocean (Kallmeyer et al., 2012). Their energy source in most of the seabed is the buried organic matter, which they oxidize to CO_2_ and inorganic nutrients. Due to the high concentration of sulfate in seawater (28 mM at an ocean salinity of 35), sulfate generally penetrates meters down into the seabed and supplies the sulfate reducing microorganisms (SRM) with an electron acceptor for their respiration. As the sediment ages with increasing burial depth beneath the seafloor, the remaining organic matter becomes steadily more refractory to microbial degradation. The time-course of organic matter degradation in the sediment, and thus of sulfate reduction rates (SRR), can be described by the sum of several exponential decay functions relating to different organic matter components, each of which is being degraded by first-order kinetics (Westrich and Berner, 1984). The sum of many such functions may be modeled as a reactive continuum (Boudreau and Ruddick, 1991) or may empirically be described by a power law function (Jørgensen, 1978; Katsev and Crowe, 2015). The latter does not have a conceptual basis similar to the reactive continuum but was found to describe experimental data on organic matter degradation rates and rate constants over a broad time interval from days to thousands of years (Middelburg, 1989; Beulig et al., 2018).

The anaerobic degradation of organic matter involves complex microbial food chains, starting with the hydrolysis of macromolecular structures by extracellular enzymes and the formation of organic molecules small enough (generally < ca. 600 dalton, but for polysaccharides possibly larger) to be taken up by bacteria or archaea (Arnosti, 2011; Reintjes et al., 2017). It is this initial hydrolysis of the complex organic material that is rate-limiting for the overall degradation rate of organic matter (Kristensen and Holmer, 2001; Arnosti, 2004; Beulig et al., 2018). Microbial cells, which take up the small organic molecules such as sugars, amino acids, lipids, organic acids etc., conserve energy and grow by multistep fermentation processes that produce a range of volatile fatty acids (VFAs), such as formate, acetate, propionate and butyrate, plus H_2_ and CO_2_.

**FIGURE 2:**
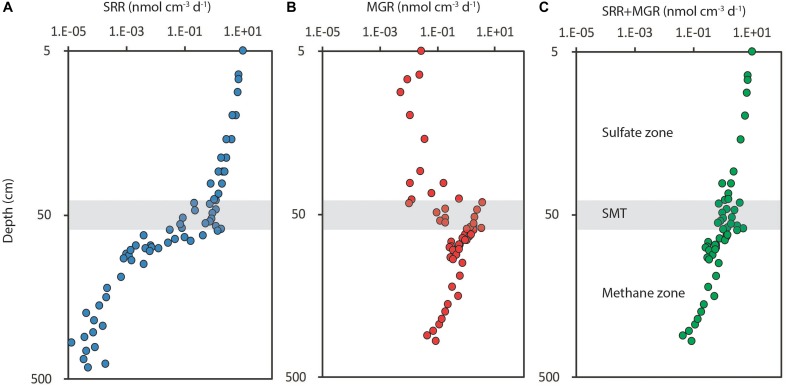
Depth distribution of organic matter degradation rates in a marine sediment from the Baltic Sea (Bornholm Basin) shown in double-log plots for the depth interval 5–500 cm. **(A)** Sulfate reduction rates (SRR), which drop off steeply where sulfate is (nearly) depleted beneath the sulfate-methane transition (SMT, gray zone at 50 cm depth); **(B)** methanogenesis rates (MGR), which are low in the sulfate zone and peak in the SMT; **(C)** sum of sulfate reduction and methanogenesis rates (SRR+MGR). Note the continuity of overall degradation rates throughout the sulfate and methane zones with only a small peak in the SMT. Redrawn from Beulig et al. (2018).

These fermentation products are used by the SRM in the downstream terminal oxidation with sulfate. When sulfate is depleted at depth, the terminal degradation in the subsurface sediment is taken over by methanogenic archaea, which have a much narrower substrate spectrum, largely restricted to H_2_/CO_2_ and potentially acetate. The metabolic rate of the SRM is limited by the production rate of their immediate substrates, which they keep at a very low threshold concentration in the low nM range for H_2_ (Hoehler et al., 1998) and in the low μM range for the VFAs (Glombitza et al., 2015). The predominant terminal process, be it iron reduction, sulfate reduction or methanogenesis, does not have a direct feed-back on the initial hydrolytic activity and, therefore, no direct effect on the overall rate of organic matter degradation, which tends to decrease in a monotonous manner throughout the sulfate and methane zones (Beulig et al., 2018; Figure 2).

### Biogeochemical Zonation

The terminal processes of organic matter mineralization display a general zonation, which reflects thermodynamic constraints on respiration using different electron acceptors combined with their sequential depletion with depth in the sediment (Froelich et al., 1979). Electron acceptors that provide higher energy yields, such as oxygen, nitrate, Mn(IV) and Fe(III), prevail near the sediment surface followed by sulfate reduction and methanogenesis. Sulfate reduction occurs also in the upper sediment layers, which are geochemically characterized by iron reduction, and the rates may even be highest in this zone (Thamdrup et al., 1994a). Yet, sulfate often shows no net depletion here due to the fast supply of seawater sulfate by bioirrigation and due to rapid re-oxidation of the produced sulfide (Canfield et al., 1993).

There is great variation in this classical zonation scheme among different ocean regions. In many deep-sea sediments the sedimentation rate and the organic carbon content are so low that sulfate penetrates all the way down to the ocean crust and prevents methanogenesis (Egger et al., 2018). In the pelagic brown and red clays underneath the Pacific gyres, mineralization is dominated by iron and manganese reduction and oxygen may penetrate very deep, even down to the basaltic crust (D’Hondt et al., 2004, 2015; Røy et al., 2012). This excludes sulfate reduction from a large part of the global seabed.

Iron reduction is limited by the reactivity of Fe(III) minerals, by the availability of electron donors, or a combination of both (Postma and Jakobsen, 1996; Thamdrup, 2000). As the sulfate reducers near the sediment surface are limited by their electron donor only, but not by sulfate, it is primarily the accessibility of iron minerals that controls the competition between iron reduction and sulfate reduction (Thamdrup et al., 1994a). Microbial iron reduction is well known from the genera *Geobacter* and *Shewanella*, but also some sulfate reducing bacteria, such as *Desulfotomaculum reducens*, are able to reduce Fe(III) in a catabolic metabolism that provides energy and supports growth (Tebo and Obraztsova, 1998; Junier et al., 2010). The contribution to iron reduction by SRM, relative to the more specialized metal reducers, remains poorly known.

### Sulfate Reduction Rates (SRR)

Sulfate reduction rates in marine sediments are determined by two main approaches: (a) transport-reaction modeling of pore water solutes (Boudreau, 1997) or (b) experimental measurements using a ^35^S-radiotracer method (Røy et al., 2014). Some models make no mechanistic assumptions about the kinetics of organic matter degradation (Berg et al., 1998; Wang et al., 2008; Lettmann et al., 2012). Other models make assumptions about the depth and age trend of mineralization rates and thereby make qualified use of information about mineralization controls and the depositional history of the sediment (Wallmann et al., 2006; Dale et al., 2008; Arndt et al., 2013). Such models may also consider the effect of bioturbation, i.e., sediment reworking and pore water advection (bioirrigation) due to burrowing macrofauna, which diminish the effect of organic matter mineralization on the pore water solute gradients (e.g., Aller and Yingst, 1985; Kristensen et al., 2012; van de Velde and Meysman, 2016).

Transport-reaction models of sulfate reduction generally assume unidirectional conversion of sulfate to sulfide. As discussed in section Sulfide Oxidation, sulfate reduction is accompanied by a concurrent sulfide oxidation driven by buried Fe(III) and other potential oxidants, thereby partly regenerating sulfate. This is particularly evident down in the “sulfate-depleted” methane zone where trace concentrations of sulfate remain (Pellerin et al., 2018a). The term “cryptic sulfur cycle” was coined for this re-oxidation – “cryptic” because it is not directly evident from the pore water chemistry (Holmkvist et al., 2011; Treude et al., 2014). As a result, there is a discrepancy between gross and net rates of sulfate reduction, the magnitude of which remains poorly constrained. Yet, sulfide oxidation and bioirrigation in surface sediments, and perhaps also enzymatic back-reaction, are likely reasons for the difference in SRR often found by modeling and by experimental rate measurements (e.g., Jørgensen and Parkes, 2010). There is a need for more detailed studies that combine these two approaches in order to understand the reason for their discrepancy. In that discrepancy may lie important information about the function of the sulfur cycle (see also section Synthesis and Future Directions).

**FIGURE 3:**
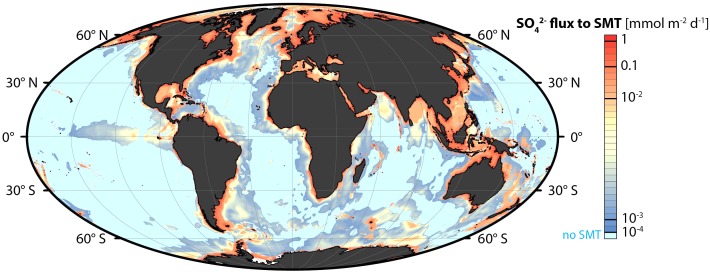
Global distribution of sulfate reduction in the seabed associated with methane oxidation and organoclastic sulfate reduction in the sulfate-methane transition (SMT) zone. The diffusive sulfate fluxes (mmol SO_4_^2-^ m^-2^ d^-1^) down into the SMT were calculated by algorithms based on pore water sulfate data from 740 sediment cores worldwide. Reproduced from Egger et al. (2018).

This methodological discrepancy becomes evident by quantitative budgets of sulfate reduction in relation to the organic carbon mineralization on local or global scales. Budgets that include both modeling and experimental rate measurements (Canfield et al., 1993, 2005; Jørgensen and Kasten, 2006), calculate significantly higher global sulfate reduction than budgets based only on diffusion-diagenesis modeling (Bowles et al., 2014). The discrepancy is particularly distinct in coastal sediments where much of the sulfate reduction takes place in the upper, bioturbated sediment and where it may therefore not be detectable as a drop in sulfate concentration in that zone. Also in the subsurface sediment, sulfate reduction is strongly focused toward the ocean margins. Based on a comprehensive database on sulfate and methane in the seabed and using environmentally calibrated algorithms for geographic extrapolation, Egger et al. (2018) developed a global map of sulfate reduction at the SMT. About 80% of the subsurface sulfate reduction was estimated to take place on the continental shelf (0–200 m water depth), which comprises only 8% of the global ocean area of 3.6⋅10^8^ km^2^, with 30% occurring within the shallowest 0–10 m (Figure 3). Canfield et al. (2005) and Jørgensen and Kasten (2006) estimated that about 70% of the global marine sulfate reduction takes place on the continental shelf. In the coastal sediments, sulfate reduction may account for half of the organic carbon mineralization in the sediment column (Jørgensen, 1982b).

Such quantitative data are important in order to understand the balance between the deposition, the anaerobic degradation, and the burial of organic matter in the seabed. The growing data frequency and geographical resolution of global maps of these processes makes it possible to analyze the environmental factors, which control the rates and the balance of the processes. The strong focusing of sulfate reduction toward the shelf and the coastal regions means that the near-surface zone of the seabed, where the highest SRR are measured, have a strong impact on the global marine sulfur cycle. This is the zone that is most affected by eutrophication and by climate change – today and in the future. It was also a highly dynamic zone in the geological past, for example as a result of the mass export of sediment and organic matter from the shelf to the continental slope, which happened during the glacial maxima (e.g., Cartapanis et al., 2016).

### Anaerobic Oxidation of Methane (AOM) With Sulfate

When sulfate is depleted at depth, methanogenesis becomes the terminal process of organic matter mineralization. It is estimated that 3–4% of the global organic carbon flux to the seafloor is converted to methane (Egger et al., 2018). As shown in Figure 2, methanogenesis rates are highest in the uppermost methane zone where the methane gradient is the steepest. Most of the methane produced in continental shelf and slope sediments therefore diffuses upwards along this gradient to meet sulfate in the SMT, where it is quantitatively oxidized by anaerobic methanotrophic archaea (ANME). Sulfate serves as the electron acceptor according to the following net equation of chemical species dissolved in the aqueous phase (e.g., Reeburgh, 2007):

(1) $${SO}_{4}^{2-}+{CH}_{4}\to {HCO}_{3}^{-}+{HS}^{-}+H_{2}O$$

The flux ratio of sulfate and methane diffusing into the SMT is often not 1:1, as predicted by the stoichiometry in Equation (1). Generally, more sulfate than methane reaches the SMT with a global mean SO_4_^2-^ to CH_4_ flux ratio of 1.4:1 (Egger et al., 2018). The 40% excess sulfate is used for organoclastic sulfate reduction by the oxidation of organic matter buried into the SMT, just as it takes place in the main sulfate zone above (Berelson et al., 2005; Burdige et al., 2016; Komada et al., 2016; Beulig et al., 2018). Methanogenesis takes over as the terminal degradation pathway well within the SMT (Figure 2). It thereby provides an additional methane source for AOM in the SMT, which is undetected by transport-reaction modeling of pore water solute gradients. By this “cryptic methane cycle” in the SMT, CH_4_ is produced and oxidized concurrently in the same sediment (Beulig et al., 2019).

Anaerobic methane-oxidizing microorganisms were first discovered as syntrophic aggregates of ANME archaea and sulfate reducing bacteria in methane- and sulfate-rich sediments (Boetius et al., 2000). Different clades of ANME are now known to form consortia with different sulfate reducing bacteria. ANME-1 and ANME-2 are usually associated with SRB of the *Desulfosarcina/Desulfococcus* branch of the Deltaproteobacteria. ANME-3 are mostly associated with SRB of the *Desulfobulbus* branch, while other ANMEs apparently do not form syntrophic aggregates (Treude et al., 2005; Knittel and Boetius, 2009). Different mechanisms have been proposed to explain the anaerobic oxidation of methane with sulfate and how the reducing equivalents are transferred from the ANME to the associated SRB. A transfer of extracellular electron carriers, such as H_2_, is thermodynamically not plausible and could not be demonstrated experimentally (Nauhaus et al., 2002). More recently, a direct interspecies electron transfer (DIET) between the ANME and the SRB cells has been suggested, possibly associated with large multiheme cytochromes detected in the SRB (McGlynn et al., 2015; Wegener et al., 2015; Skennerton et al., 2017). Such a DIET was supported by the observation that electrons from ANME during methane oxidation may be transferred to artificial electron acceptors instead of to SRB (Scheller et al., 2016).

### Sulfate Reducing Microorganisms (SRM)

The sulfate reducers comprise a very diverse group of anaerobic microorganisms, mostly of the Bacteria domain, with catabolic capacities for a wide spectrum of fermentation products. These include primarily H_2_ and VFAs but also many other substrates such as hydrocarbons or aromatic compounds. Many SRM belong to the Deltaproteobacteria, including members of the *Desulfovibrionales* and *Desulfobacterales* orders. The *Desulfotomaculum* are Gram-positive bacteria, characterized by the ability to form endospores. The SRM found in marine sediments mostly belong to uncultured groups that are only distantly related to cultivated sulfate reducers. Among the abundant SRM, some of which do have cultured relatives, are the deltaproteobacteria *Desulfobacteraceae* (in particular from the *Desulfococcus* and *Desulfosarcina* cluster) and *Desulfobulbaceae*. Deeper in the sediments, other taxa of SRM become predominant, such as the phyla Firmicutes, Chloroflexi, and Atribacteria (Leloup et al., 2009; Carr et al., 2015; Wasmund et al., 2017). Recent genomic data from marine and terrestrial subsurface environments have revealed the potential capacity for sulfate or sulfite reduction in many other bacterial and archaeal phyla that were not previously associated with this process (Anantharaman et al., 2018). The functional significance of this broad diversity of SRM for the marine sulfur cycle is currently not known.

**FIGURE 4:**
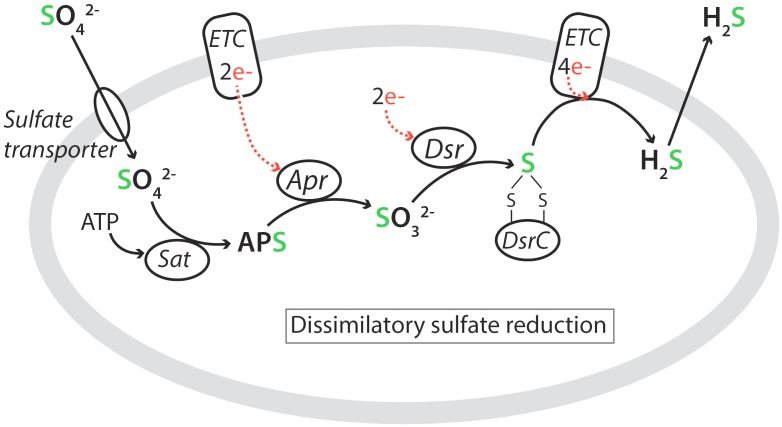
Metabolic pathway of dissimilatory sulfate reduction showing the active uptake of SO_4_^2-^ by a membrane-bound sulfate transporter, the four steps in the reduction pathway, and the passive release of H_2_S (see text). Abbreviations: Sat, ATP sulfurylase; APS, adenosine-5′-phosphosulfate; Apr, adenylyl-sulfate reductase; Dsr, dissimilatory (bi)sulfite reductase; ETC, membrane-bound electron transfer complex. Sulfur is green while electron transfers to sulfur are indicated in red. After Santos et al. (2015) and Sim et al. (2017).

The known SRM share a common pathway for DSR, which is illustrated in Figure 4 (Rabus et al., 2015; Santos et al., 2015). Sulfate is taken up from the environment by low- or high-affinity sulfate transporters and becomes activated with ATP in the cytoplasm by the enzyme ATP sulfurylase (Sat) to form adenosine-5′-phosphosulfate (APS). The APS is reduced to sulfite by adenylyl-sulfate reductase (Apr), which receives electrons from a membrane-bound electron transfer complex (ETC). The (bi)sulfite is further reduced to H_2_S by the dissimilatory (bi)sulfite reductase (Dsr) complex via a DsrC-bound trisulfide (Santos et al., 2015). The produced H_2_S diffuses passively out through the cell membrane.

While this is the main forward direction of microbial sulfate reduction, each step has a certain reversibility determined by the intermediate substrate and product concentrations, which together generate the forward thermodynamic drive. This enables a partial back-reaction, which provides a mechanism for sulfur isotope fractionation (e.g., Wing and Halevy, 2014; Sim et al., 2017) (see section Stable Sulfur Isotopes).

Functional marker genes for the key enzymes of DSR are used to study the diversity of SRM (Wagner et al., 2005; Müller et al., 2014) and to determine their distribution and abundance in the environment. The SRM communities in the upper, bioturbated zone of the seabed differ distinctly from the deeper subsurface communities. For example, in studies from Aarhus Bay, between the Baltic Sea and the North Sea, the microbial communities, including the SRM, were found to have high diversity within the upper 5–10 cm of bioturbated sediment (Jochum et al., 2017; Petro et al., 2017). The diversity decreased with depth and age in the sediment, concurrently with a shift of the total community from strong predominance of Bacteria to nearly equal abundance of Bacteria and Archaea at depth (Chen et al., 2017). Importantly, the assembly of the subsurface communities, i.e., the establishment of their diversity and community structure, was found to take place at the base of the bioturbated zone, below which rare community members from the surface sediment persisted and became dominant as the community was slowly buried and became isolated. The genetic and physiological diversity of the subsurface communities was thus a result of purifying selection rather than of mutation (Starnawski et al., 2017). Such a purifying selection implies a gradual loss, through many generations, of the less competitive species and increasing dominance of the more competitive species under the environmental conditions in the subsurface sediments. The resulting reduction in species richness of microbial communities may continue for hundreds of thousands of years as the sediment is steadily buried deeper (Walsh et al., 2016).

SRM are distributed through all biogeochemical zones in the seabed, from the heterogeneous and chemically fluctuating surface sediment throughout the sulfate zone and deep into the sulfate-depleted methane zone (Gittel et al., 2008; Leloup et al., 2009; Orsi et al., 2016; Jochum et al., 2017). The general abundance of microorganisms decreases with depth and age in the sediment (Kallmeyer et al., 2012; Parkes et al., 2014; Jørgensen and Marshall, 2016) and so does the number of SRM cells. This was shown in extracted DNA by targeting diagnostic single-copy genes such as those encoding for the alpha or beta subunit of dissimilatory sulfite reductase (*dsrAB*). The decline in abundance of SRM is even steeper than that of the total microbial community. In the top 5–10 cm of sediment, which constitutes a heterogeneous and variable environment due to mixing (bioturbation) and irrigation by burrowing macrofauna, the SRM may apparently comprise up to 25% of all microbial cells, while down through the sulfate zone this number gradually drops below 5% and approaches 2–3% at depth (Jochum et al., 2017). The relative abundance of SRM is elevated in the SMT where the community feeds on methane in addition to the buried organic matter. In the methane zone, SRM are also present, but in low numbers. The relative SRM abundances cited here for subsurface sediment are lower than data obtained for the same sediments a decade earlier by Leloup et al. (2009). The difference may be ascribed to new DNA extraction methods (Lever et al., 2015) and to a larger diagnostic gene sequence database for SRM, which has led to more specific qPCR primers for *dsrB* gene quantification (Müller et al., 2014; Jochum et al., 2017).

### Controls on SRM Communities

**FIGURE 5:**
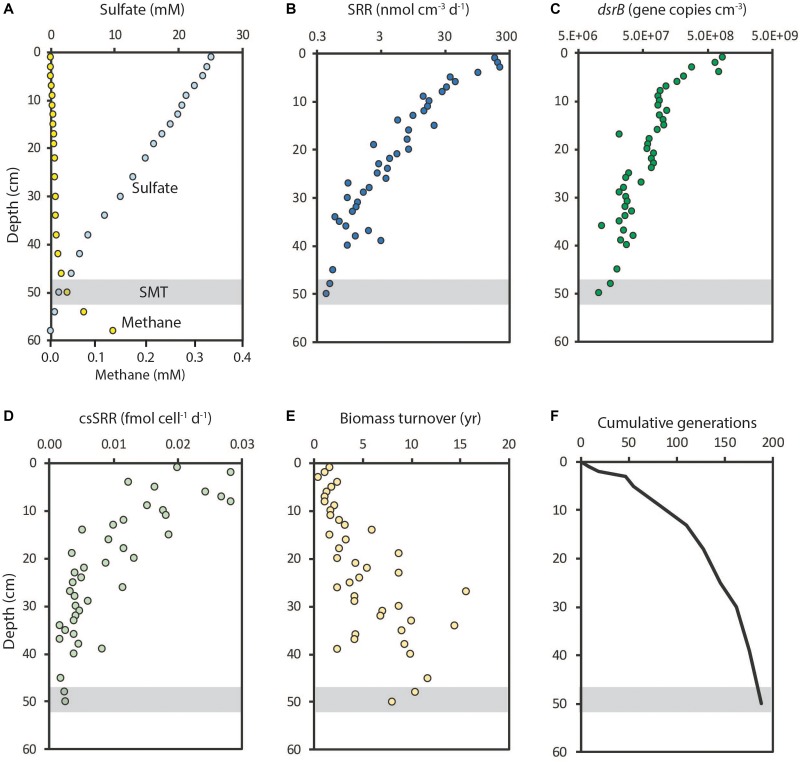
Depth distributions of sulfate reduction and sulfate reducers at Station M5, Aarhus Bay. **(A)** Sulfate and methane concentrations; a sulfate-methane transition (SMT) was located at 50 cm depth. **(B)** Sulfate reduction rates from ^35^SO_4_^2-^ experiments. **(C)** Abundance of sulfate reducing microorganisms (SRM) determined from *dsrB* gene copies. **(D)** Mean cell-specific sulfate reduction rates (csSRR). **(E)** Estimated biomass turnover time (years) of sulfate reducers. **(F)** Cumulative generations of SRM during burial. Data from Petro et al. (2019).

The abundance of SRM in the sulfate zone of marine sediments is related to the availability of electron donors and sulfate and, thus, to the potential for anaerobic respiration. Figure 5 shows a case study from Aarhus Bay where sulfate penetrated to about 50 cm sediment depth below which methane accumulated (Petro et al., 2019). Experimental measurements of sulfate reduction showed that rates dropped by 500-fold with depth from the bioturbated surface sediment and down through the sulfate zone where the organic matter became increasingly recalcitrant with increasing age of the sediment (e.g., Middelburg, 1989; Figure 5B). The SRM abundance, determined from the *dsrB* gene copy numbers, dropped off by only 50-fold over the same depth interval (Figure 5C). The mean SRR per cell (cell-specific SRR or csSRR), calculated from the SRR divided by the SRM abundance, thus dropped 10-fold, from 0.03 to 0.003 fmol SO_4_^2-^ cell^-1^ day^-1^ (1 fmol = 10^-15^ mol) (Figure 5D). For comparison, pure cultures of SRM under laboratory conditions have 1000-fold higher mean SRR per cell, in the order of 1–10 fmol SO_4_^2-^ cell^-1^ day^-1^ at psychrophilic temperatures and 5–50 fmol SO_4_^2-^ cell^-1^ day^-1^ at mesophilic temperatures (Knoblauch and Jørgensen, 1999; Knoblauch et al., 1999; Detmers et al., 2001; Tarpgaard et al., 2006).

Even much lower cell-specific SRR have been calculated for deep sub-seafloor communities. This raises the question about the minimum energy turnover needed to maintain the SRM community (their “basal power requirement,” according to Hoehler and Jørgensen, 2013). Recent data indicate that this power requirement is higher for sulfate respiring cells than it is for the great majority of fermenting microorganisms (Marion Jaussi and Hans Røy, personal communication). The reason for this is not known but it could reflect a higher energetic maintenance cost of anaerobic respiration than of fermentation. However, pure culture data compiled by LaRowe and Amend (2015) relating cell-specific maintenance power requirements to metabolic pathway showed large variations but no clear pattern.

It remains an open question whether the very low cell-specific SRR provides enough energy to also enable growth of the sulfate-reducing cells. If one assumes a mean cell biomass of 20 fg C (Braun et al., 2016; 1 fg = 10^-15^ g) and a hypothetical growth yield of 8% (Petro et al., 2019), then the mean biomass turnover can be calculated (Lomstein et al., 2012). The mean biomass turnover time in Aarhus Bay increased from a few years near the sediment surface to more than 10 years at 50 cm depth (Figure 5E). This means that the biomass turnover enabled only about 200 generations of microorganisms during the 500 years of burial from the sediment surface to 50 cm depth (Figure 5F). This limited number of generations explains why there was little capacity for mutational change during burial of the community (Starnawski et al., 2017). It should be noted that, if the actual mean growth yield is lower than 8%, then the turnover time is correspondingly longer and the number of generations correspondingly lower.

Several studies have analyzed which substrates play a quantitative role as electron donors for SRM in marine sediments. Radiotracer experiments with ^14^C-labeled substrates have shown acetate to be the main fermentation product feeding the SRM (Christensen and Blackburn, 1982; Shaw and McIntosh, 1990; Beulig et al., 2018). Experiments with marine sediment from Aarhus Bay and from a Svalbard fjord based on a specific inhibition of sulfate reduction by molybdate or selenate indicated the following substrate (electron donor) contributions to sulfate reduction: 40–50% acetate, 10–20% propionate, 10% butyrate, and 5–10% H_2_ plus several minor substrates (Sørensen et al., 1981; Finke et al., 2007).

The efficiency of substrate uptake by the SRM generally controls the pore water concentrations of VFAs and H_2_ beneath the bioturbated zone. Yet, the substrate availability for the SRM is determined by the production rate of useable fermentation products, rather than by their concentration. Even at relatively high organic matter turnover, the H_2_ concentration is maintained at a few nM (Hoehler et al., 1998) while the VFAs are maintained at low μM level (Glombitza et al., 2015).

**FIGURE 6:**
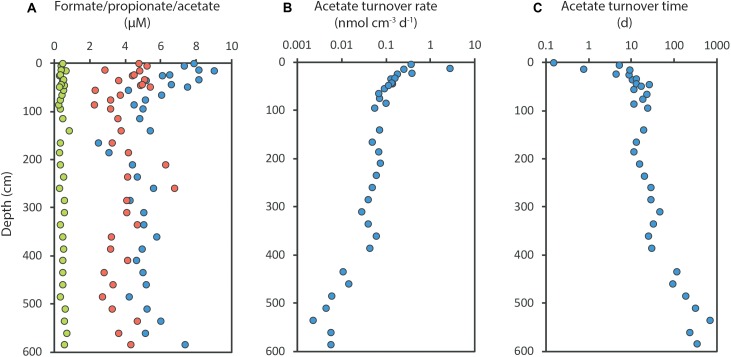
Depth distributions of volatile fatty acids and acetate turnover in a 600 cm deep sediment core from the arctic shelf off Southwest Greenland. **(A)** Formate (red), propionate (green) and acetate (blue) concentrations; **(B)** Acetate turnover rates; **(C)** Acetate turnover time. Redrawn from Glombitza et al. (2015).

As an example of this from the continental shelf off West Greenland, the SRR decreased by more than 1000-fold down through a 600 cm deep sediment column, yet the VFA concentrations remained very constant: 4–9 μM acetate, 2–5 μM formate, and 0.3–0.7 μM propionate (Glombitza et al., 2015; Figure 6A). The sulfate reduction rate, and thus the acetate turnover rate, dropped steeply with depth (Figure 6B), while the acetate concentration did not change significantly. The turnover time of acetate increased from 10 h near the sediment surface to 4 years at 600 cm depth (Figure 6C). The acetate turnover was thus extremely slow in the deep sediment, yet the calculated mean diffusion time of acetate between cells was less than 1 s, even at 600 cm depth. The subsurface cells are therefore living in a highly stable environment with uniformly low substrate concentration.

Considering the extreme range of turnover times, it is not clear why the substrate concentrations remain so constant and why the substrates are not depleted further. For comparison, a chemostat-grown culture of the acetate-oxidizing SRM, *Desulfobacter postgatei*, had a rather high half-saturation constant (apparent *Km*) of 70 μM acetate, but in the resting stage the organisms depleted the acetate concentration to <1 μM (Ingvorsen et al., 1984). The Gibbs energy (ΔG*_r_*) for acetate-utilizing sulfate reduction in the Greenland sediment dropped across the 0–600 cm depth interval from -45 kJ mol^-1^ near the sediment surface to -31 kJ mol^-1^ at depth. Although this may not signal strong thermodynamic control (Glombitza et al., 2015) laboratory experiments with continuous pure cultures are required to understand how such threshold substrate concentrations may be energetically controlled. The corresponding drop in SRR was from about 1 to 0.01 nmol SO_4_^2-^ cm^-3^ d^-1^. The power available to the microorganisms can thus be calculated from the product of the Gibbs energy and the SRR (cf. LaRowe and Amend, 2015). This volume-specific power supply, P*_s_*, dropped from 5 × 10^-10^ W cm^-3^ at the sediment surface to 4 × 10^-12^ W cm^-3^ at 600 cm depth. This is within a range typical of marine shelf sediments (LaRowe and Amend, 2015).

Sulfate reduction rates are also dependent on the availability of sulfate. Experiments with sulfate-depleted marine sediment have indicated that sulfate may become limiting at low mM concentrations with an apparent half-saturation constant (*Km*) of 0.1–3 mM (Boudreau and Westrich, 1984; Roychoudhury et al., 2003; Pallud and Van Cappellen, 2006). This rather high *Km* appears to contradict the general observation of a peak in sulfate reduction rate within the SMT where the sulfate concentration is very low and sulfate reduction is mainly fueled by methane, which is energetically a poor substrate (Holler et al., 2011). Experiments with marine sediment have more recently shown that the SRM community can shift the apparent *Km*, depending on the availability of sulfate (Tarpgaard et al., 2011). At high sulfate concentration, the sulfate uptake by the SRM in a marine sediment had low affinity (*Km* = 0.4 mM), while at low sulfate concentration, the SRM switched to high-affinity sulfate uptake (*Km* = 0.003 mM). The active sulfate transporters responsible for this shift in affinity are poorly know. However, a previously overlooked group of CysZ-type putative sulfate transporters was recently suggested to play a key role for the high-affinity sulfate uptake (Marietou et al., 2018).

It was not known whether the results of Tarpgaard et al. (2011) from marine sediment reflected a switch between different populations of sulfate reducers, some with low and some with high sulfate affinity. Pure culture experiments showed later that the marine sulfate reducer, *Desulfobacterium autotrophicum*, can up-regulate a high-affinity sulfate uptake system and thereby switch the apparent *Km* from 0.5 to 0.008 mM when the external sulfate concentration drops below 0.5 mM (Tarpgaard et al., 2017). Recent data show that the “cryptic sulfur cycle” in the methane zone is operating at steady state sulfate concentrations of 0.01 mM or less (Pellerin et al., 2018a). Such sulfate concentrations are apparently balanced between slow production from reaction of sulfide with buried Fe(III) and slow consumption by the SRM. The low sulfate concentrations may represent an energetic minimum threshold for sulfate uptake under the available conditions.

## Sulfide Oxidation

**FIGURE 7:**
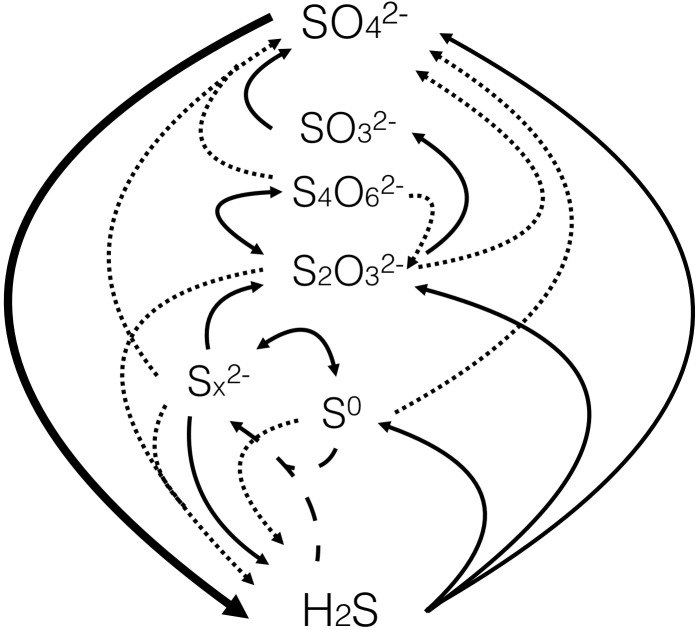
Overview of the processes and major inorganic species of the sulfur cycle. The large black arrow represents sulfate reduction, thin black lines represent oxidation and dotted lines represent disproportionation reactions. The dashed lines represent equilibration to form polysulfides. Schematic adapted from Zopfi et al. (2004).

Mass balance estimates and diffusion gradients of sulfide indicate that a significant fraction of the sulfide produced by sulfate reduction in marine sediments is reoxidized (Jørgensen, 1982b; Canfield et al., 1992; Pellerin et al., 2015b). This reoxidation occurs through diverse biological and geochemical pathways, forming a variety of reactive intermediates (Figure 7). The extent of sulfide reoxidation depends upon the quantity and type of available oxidant as well as the presence of microorganisms (e.g., Luther et al., 2011).

### The Dynamic Surface Sediment

In most coastal sediments, oxygen is depleted within the surface millimeters (Revsbech et al., 1980), leaving the remaining 3–10 cm deep surface sediment partially oxidized, but anoxic. When oxygen is depleted nitrate, Mn oxides and Fe oxides are the next most important oxidants present in the sediment and are typically depleted in this order due to coupling with organic matter oxidation, as previously discussed. In addition to oxidizing organic matter, however, these species are also oxidants for sulfide. This is exemplified by the presence of a gap between detectable oxygen and sulfide concentrations (a “suboxic zone”), commonly of several centimeters thickness in many coastal sediments. Despite the lack of detectable sulfide in this zone, high SRR can be measured within the zone, meaning that sulfide is indeed produced, but rapidly reoxidized. For example, SRR of 32 nmol cm^-3^ d^-1^ were measured in Aarhus Bay in the surface sediment, where sulfide was not detectable (≤1 μM). In this case, the sulfide turnover time was less than 30 min, possibly much less (Thamdrup et al., 1994a). In such anoxic sediments, Mn oxides, and particularly Fe oxides, are the dominant chemical oxidants for sulfide, while a portion of the sulfide oxidation is microbially mediated with nitrate (Jørgensen and Nelson, 2004).

Microorganisms are capable of catalyzing sulfide oxidation at rates that are orders of magnitude higher than the chemical oxidation, depending upon the biogeochemical characteristics of the sediment or water column (Jørgensen, 1982a; Luther et al., 2011). Moreover, bacteria capable of oxidizing sulfide and metabolizing other sulfur compounds are diverse and prevalent in the environment. Sulfide oxidation occurs most intensively in surface sediment due to the high rates of sulfate reduction and the high availability of oxidants to which they can couple the oxidation of sulfide (Canfield, 1989; Thamdrup et al., 1994a,b). The most well-characterized sulfide oxidizing bacteria in laboratory cultures also originate mostly from surface sediments. In the bulk sediment below, it is less clear which bacteria are responsible for sulfide oxidation or what their relative contribution is (cf. Wasmund et al., 2017).

Analyses of 16S rRNA and functional marker genes in amplicon and metagenomic data reveal a large diversity of potentially sulfide oxidizing microorganisms in sediments. Of the cultivated genera, such as *Thiobacillus* and *Thiomicrospira*, many are autotrophic or mixotrophic and couple the oxidation of sulfide with chemoautotrophic CO_2_ assimilation. Yet, these genera do not appear to be the predominant, active sulfide oxidizers in marine sediments (e.g., Brinkhoff et al., 1998). Experiments with non-phototrophic CO_2_ assimilation in sediments have used ^14^C-microautoradiography (Lenk et al., 2011) or ^13^C incorporation into bacterial phospholipid fatty acids (PLFA) (Boschker et al., 2014) to identify which cells are involved in dark, sulfide-dependent CO_2_ fixation. By combination of ^14^CO_2_ assimilation and gene analyses, uncultured *Gammaproteobacteria* were suggested to play the most important role (Dyksma et al., 2016) and to constitute 40–70% of the CO_2_-fixing sulfide oxidizers (Lenk et al., 2011). Boschker et al. (2014) found that dark CO_2_ fixation corresponded to 15–30% of the sediment oxygen uptake in a coastal sediment, which would suggest an extremely high growth yield of the sulfide oxidizing bacteria, even higher than that found in pure cultures (Nelson et al., 1986; Jørgensen and Nelson, 2004).

In addition to the oxidation of sulfide, bacteria are clearly involved in the turnover of intermediate sulfur species, based both on genetic characterization (Wasmund et al., 2017) and experimental results from environmental samples (Zopfi et al., 2004; Findlay and Kamyshny, 2017). For example, many SRM can also disproportionate elemental sulfur and thiosulfate (Bak and Pfennig, 1987; Kramer and Cypionka, 1989). The advantage to using intermediate sulfur species is that many of these compounds have high redox potential and, in the case of thiosulfate or sulfite, must not be activated with ATP, in contrast to sulfate.

For further discussion of microbial sulfide oxidation, including relevant insights from metagenomics and 16S rRNA studies, the reader is referred to the recent review of Wasmund et al. (2017).

### The Specialist Sulfide Oxidizers

The most conspicuous microorganisms responsible for sulfide oxidation are the large specialist sulfide oxidizers of the gammaproteobacterial family *Beggiatoaceae*, such as the filamentous *Beggiatoa* and *Thioploca* or the spherical *Thiomargarita* (Jørgensen and Nelson, 2004; Dale et al., 2009; Salman et al., 2013). It should be noted that, through single-cell sequencing of morphologically identified sulfur bacteria, this taxonomy was revised by Salman et al. (2011) who proposed to divide the family *Beggiatoaceae* into seven new *Candidatus* genera. These large bacteria are generally limited to the surface layer of organic-rich sediments where they can utilize steep chemical gradients of sulfide and oxidants (nitrate and oxygen).

The bacteria have developed interesting adaptations to bridge the spatial or temporal gap between sulfide and oxidants, such as motility and storage of nitrate and elemental sulfur (Schulz and Jørgensen, 2001). The nitrate is stored in vacuoles in up to several hundred mM concentration and may support cellular respiration for days to months. Elemental sulfur, formed as an intermediate during sulfide oxidation, is stored in membrane invaginations in the cytoplasm and serves as an energy-rich electron donor for, similarly, long periods. The filaments glide up and down in the several-cm thick, seemingly oxidized surface sediment by random (*Beggiatoa*; Dunker et al., 2011) or oriented (*Thioploca*; Jørgensen and Gallardo, 1999) patterns of movement. *Thiomargarita*, in contrast, is practically immotile, but the extremely large cells of several hundred μm diameter have sufficient storage capacity to endure starvation from sulfide or nitrate for months (Schulz et al., 1999).

It was discovered only recently that this same ecological niche is used also by several-centimeter long chains consisting of hundreds to thousands of bacteria, now called cable bacteria (Nielsen and Risgaard-Petersen, 2015). These bacteria span the vertical gap between sulfide and oxygen in the uppermost few cm of many sulfide-rich coastal sediments. Interestingly, they separate the two half reactions in the redox process of sulfide oxidation so that the main subsurface part of the cable oxidizes sulfide without immediate access to an oxidant. Instead, the electrons from sulfide are conducted up through the cable, apparently via multiple, continuous periplasmic strings, to reach the top of the cable, which transfers the electrons to oxygen, thereby completing the redox process of aerobic sulfide oxidation (Pfeffer et al., 2012; Bjerg et al., 2018). The electron transfer to oxygen consumes protons and thereby generates a distinct pH peak at the oxic-anoxic interface (Equation 2). Since most other oxidation processes at this interface tend to lower the pH, a pH peak is a strong indicator that cable bacteria are active (Nielsen et al., 2010; Risgaard-Petersen et al., 2012; Meysman et al., 2015):

(2) $$O_{2}+{4e}^{-}+{4H}^{+}\to 2H_{2}O$$

Cable bacteria may also use nitrate as electron acceptor (Marzocchi et al., 2014). Their community size may grow to more than a kilometer of filaments (nearly 10^9^ cells) per cm^2^ and thereby compete effectively wiatoa (Schauer et al., 2014). By oxidizing reduced sulfur and iron in the surface sediment they may prevent or delay the release of sulfide during periods of bottom water anoxia in coastal waters (Seitaj et al., 2015). The candidate genera names *Electrothrix* and *Electronema* were proposed for the identified bacteria, which are classified within the deltaproteobacteria, *Desulfobulbaceae*, the members of which are otherwise known to be sulfate reducers (Trojan et al., 2016). Cable bacteria are presently not know to perform DSR.

The gap between oxygen and sulfide in porous, coastal sediments may also be inhabited by chemoautotrophic bacteria living inside invertebrates, such as gutless oligochaetes or nematodes (Dubilier et al., 2008). Most of these symbiotic bacteria are chemoautotrophic sulfide oxidizers and are transported around in the surface sediment inside their meiofauna hosts.

### Reactions of Sulfide With Fe and Mn Minerals

Bioturbation by macrofauna maintains iron and manganese in the oxidized state and thereby enhances the sulfide oxidation potential completely to sulfate (Aller, 1994a,b). Below the bioturbated zone, low rates of sulfide oxidation may be sustained by slow reaction with poorly reactive iron minerals. This oxidation may occur on timescales of hundreds to hundreds of thousands of years (Canfield et al., 1992; Holmkvist et al., 2011), and sulfide oxidation is expected to be incomplete. The presence of turbidites within the sulfide zone also causes local non-steady-state conditions that lead to enhanced sulfide oxidation within these layers (Yücel et al., 2010). Sulfide oxidation can also occur below the SMT or below the sulfide zone in some sediments, due to the presence of reactive iron in the underlying lacustrine sediment (e.g., Holmkvist et al., 2011, 2014; Pellerin et al., 2018a). Much of what is known about microbial sulfide oxidation comes from studies of bacteria found in surface sediment, however, and much less is known about the potential for microbial sulfide oxidation in the deeper sediment layers.

In the literature, “reactive iron” refers to iron that may react with sulfide over timescales from seconds to thousands of years (e.g., ferrihydrite, goethite, hematite; Canfield, 1989). Iron speciation in sediments is heterogeneous and the reactivity of those Fe minerals considered very reactive toward sulfide varies over at least two orders of magnitude (Poulton et al., 2004). Regardless of iron speciation, Fe(III) (oxyhydr)oxides react with sulfide via a surface controlled reaction in which the sulfide is adsorbed onto the mineral surface and is oxidized via an inner-sphere orbital electron transfer (Luther, 1990). Dos Santos Afonso and Stumm (1992) proposed a one-electron transfer to form an S radical, which can then react further with iron to form sulfate. In their study, which was conducted in 0.1 M NaClO_4_, sulfate and thiosulfate were observed to be the major products of the reaction. Other experimental determinations of reaction products in seawater or artificial seawater have, however, led to differing results, with elemental sulfur being formed as the dominant product (Pyzik and Sommer, 1981; Yao and Millero, 1996; Poulton et al., 2004), possibly through a polysulfide intermediate (Wan et al., 2014). The observation of elemental sulfur as the dominant product of abiotic sulfide oxidation with Fe(III) is consistent with the underlying inorganic chemical theory (Luther, 1990). This reaction can be quite fast, so that iron minerals are reduced by sulfide even in sediments in which heterotrophic iron reduction also occurs, leading to competition between chemical and biological processes (Canfield, 1989; Hansel et al., 2015).

Manganese oxides are stronger oxidants for sulfide than iron oxides and the reaction occurs correspondingly faster (Yao and Millero, 1993, 1996). As for iron, the reaction rate is also dependent upon the speciation of manganese. Freshly precipitated δMnO_2_ is for example an order of magnitude more reactive to sulfide than aged δMnO_2_ or γMnOOH (Yao and Millero, 1993). Moreover, although a two-electron oxidation of sulfide to elemental sulfur is predicted for this reaction (Burdige and Nealson, 1986; Yao and Millero, 1996), sulfide oxidation by MnO_2_ has consistently been shown to produce oxidation products of higher oxidation state, such as thiosulfate and even sulfate (Aller and Rude, 1988; Böttcher and Thamdrup, 2001), also under purely abiotic conditions (Yao and Millero, 1996). Although in natural systems microorganisms can affect the products, there may also be a dependence upon pH, with sulfate as the dominant product at low pH and elemental sulfur as the main product (80%) at pH 8 (Herszage and dos Santos, 2003).

In addition to the inorganic reactions with iron and manganese minerals, it is possible that reaction of sulfide with organic compounds could be significant, forming in addition to organic sulfur also elemental sulfur and thiosulfate. At pH 6, dissolved organic matter (humic acids) reacts with sulfide at rates comparable to the reaction between sulfide and poorly crystalline iron oxides to form elemental sulfur and thiosulfate (Heitmann and Blodau, 2006; Yu et al., 2015). It remains unknown whether this process is significant in marine sediments.

### Dynamics of Intermediate Sulfur Species

The prevailing products of sulfide oxidation vary, depending upon whether the process is biotic or abiotic, the type of oxidant and the oxidant-to-sulfide ratio. However, the prevailing inorganic intermediate sulfur species are polysulfides, elemental sulfur, thiosulfate, sulfite and tetrathionate.

Elemental sulfur is not formed during sulfate reduction but is an important intermediate of sulfide oxidation. It is meta-stable and typically present in marine sediments in relatively high concentrations, compared to the more oxidized anions (thiosulfate, sulfite, tetrathionate; Troelsen and Jørgensen, 1982; Thamdrup et al., 1994b; Zopfi et al., 2004). Although elemental sulfur is typically defined operationally (e.g., solid-phase elemental sulfur extractable by organic solvents such as methanol or toluene), recent work indicates that the speciation and reactivity of elemental sulfur is heterogeneous, and that this heterogeneity may have biogeochemical implications. For example, nanoparticulate elemental sulfur with a particle size <0.2 μm has recently been detected in a variety of environments, including sediment pore waters (Findlay et al., 2014; Pellerin et al., 2018a). Moreover, sulfur produced microbially during sulfide oxidation has a diversity of forms, depending upon the microorganism (Steudel et al., 1988, 1990; Prange et al., 2002; Kleinjan et al., 2003). The reactivity of this biological sulfur diverges from that of inorganic α-S_8_ with respect to both geochemical reactions (e.g., Kamyshny and Ferdelman, 2010; Holmkvist et al., 2011; Lichtschlag et al., 2013; Garcia and Druschel, 2014) and microbial metabolism (Franz et al., 2007; Findlay and Kamyshny, 2017). More recently, elemental sulfur encapsulated in microstructures of organic matter has been observed to form along oxygen/sulfide gradients in laboratory experiments. They are likely present in the environment as well (Cosmidis and Templeton, 2016).

Elemental sulfur reacts with sulfide to form polysulfides, which are reactive in a variety of biogeochemical processes (for review see Findlay, 2016). The processes are affected by the speciation of elemental sulfur (e.g., Kleinjan et al., 2005), which may lead to a discrepancy between polysulfide concentrations expected from thermodynamic equilibrium between sulfide and elemental sulfur and the actual concentrations observed in the environment (Kamyshny and Ferdelman, 2010; Lichtschlag et al., 2013; Holmkvist et al., 2014). Polysulfides have a particularly important role for pyrite formation in marine sediments (section Formation of Pyrite, cf. Rickard and Luther, 2007).

**FIGURE 8:**
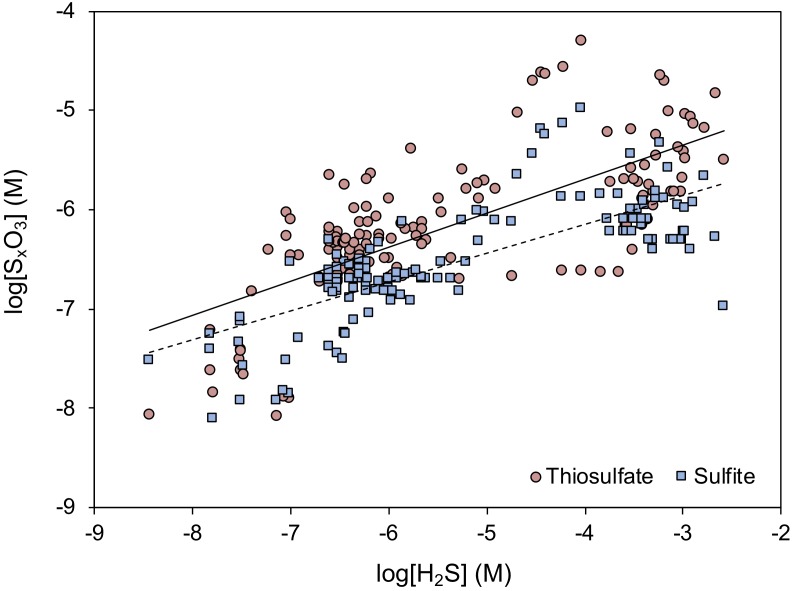
Compiled relationship between thiosulfate (red circles) and sulfite (blue squares) concentrations in marine sediments in relation to the ambient concentration of free sulfide. Redrawn after Blonder et al. (2017). Log-log linear regressions are shown for thiosulfate (full line; *R*^2^ = 0.51) and sulfite (broken line; *R*^2^ = 0.57). For a complete discussion regarding the individual data points, the reader is referred to the original article.

Of the major oxyanions, all are typically present at low, micromolar or sub-micromolar concentrations, controlled by their rapid turnover (Zopfi et al., 2004; Findlay and Kamyshny, 2017). Interestingly, the presence and concentration of intermediate sulfur species appear not to correlate directly to sulfide concentrations (Figure 8), perhaps due to the opposing controls of formation and consumption rates. With increasing sulfide concentration, the oxidant-to-sulfide ratio tends to decrease and the formation rate of thiosulfate and sulfite decreases correspondingly. At the same time, the concentrations of thiosulfate and sulfite decrease, which coincides with a decrease in the consumption rates of these species (Blonder et al., 2017). Sulfite is more reactive than thiosulfate, also in abiotic reactions (e.g., by sulfurization of organic matter), whereas thiosulfate appears to be chemically stable in sediments and is microbially consumed. This consumption proceeds through reduction, oxidation or disproportionation, all of which may occur simultaneously in surface sediments (Jørgensen and Bak, 1991).

Tetrathionate (S_4_O_6_^2-^) is also readily used by microorganisms in marine sediments (Zopfi et al., 2004; Findlay and Kamyshny, 2017) and may be important in some environments (Podgorsek and Imhoff, 1999), but tetrathionate is rarely observed because concentrations are typically below detection (<0.5 μM; Zopfi et al., 2004). Yet, it has a high redox value and is readily utilized by a diverse array of microorganisms (Barrett and Clark, 1987). The reduction of tetrathionate does not appear to be connected to the oxidation of organic matter (Zopfi et al., 2004), and it is not clear what the role of microbial tetrathionate metabolism is in the environment.

### Potential for Sulfate Formation During Sulfide Oxidation

The formation of intermediate sulfur species is observed when sulfide oxidation is studied under experimental conditions in which the immediate oxidation products can be stabilized and measured. They are also detectable at low concentrations in natural systems. However, sulfate is the stable oxidized end-member, and pathways for sulfate formation in anoxic sediments are therefore important, yet unclear. There are two main possibilities: (1) direct chemical or microbial oxidation of sulfide to sulfate (through intermediate species) and (2) microbial disproportionation of intermediate species (elemental sulfur, thiosulfate or sulfite) formed from the partial oxidation of sulfide.

As discussed above, at circum-neutral pH the inorganic oxidation of sulfide by both iron and manganese oxides appears to result mainly in the formation of elemental sulfur. A complete oxidation of sulfide to sulfate with manganese oxides may take place abiotically but can also be microbially mediated (Schippers and Jørgensen, 2001). This may be particularly important in the more oxidized surface sediments in which oxidant concentrations are much higher than sulfide concentrations. Indeed this was observed in amendment experiments in which Mn and Fe oxides were added to sulfidic sediment. Addition of Mn(IV) resulted in significant sulfate formation, whereas addition of Fe(III) had little effect on sulfate production (Aller and Rude, 1988). Similar results were achieved in culture experiments with sulfur disproportionating bacteria. In the presence of manganese oxides significant oxidation of sulfide to sulfate occurred without disproportionation (Böttcher and Thamdrup, 2001). In the presence of iron oxides, microbial disproportionation was necessary in order to form sulfate (Böttcher et al., 2001).

Bacteria capable of disproportionating intermediate sulfur species are widespread in marine surface sediments (Bak and Pfennig, 1987; Thamdrup et al., 1993; Finster et al., 1998). As the ratio between sulfide and potential oxidants decreases, elemental sulfur should be the more prevalent intermediate formed. This may inhibit complete oxidation, as elemental sulfur disproportionation becomes thermodynamically unfavorable at free sulfide concentrations greater than ca 1 mM. Under such high sulfide concentrations, however, polysulfides form and these may also be disproportionated. The thermodynamics of polysulfide disproportionation appear to be less sensitive to sulfide concentrations than of elemental sulfur (Milucka et al., 2012; Poser et al., 2013). Thiosulfate disproportionation does not appear to be sensitive to sulfide concentration (Jørgensen and Bak, 1991), but in very sulfidic sediment, thiosulfate is not expected to be a major product of sulfide oxidation (e.g., Blonder et al., 2017).

### Determination of Sulfide Oxidation

In contrast to sulfate reduction rate measurements using ^35^S-radiotracer, the experimental quantification of sulfide oxidation by the use of ^35^S-labeled sulfide is complicated by rapid isotope exchange, which takes place between sulfide, elemental sulfur, polysulfide and FeS (Fossing and Jørgensen, 1990a; Fossing et al., 1992). Isotope exchange involves the interchange of sulfur atoms between different species without a net transfer of mass (e.g., through oxidation or reduction) between the involved species. Therefore, an observed transfer of radioactivity is deceiving as it may not imply a net transformation of the compound itself. Importantly, such isotope exchange has not been detected between sulfate and the reduced sulfur species under sediment conditions. It is interesting to note that, in spite of the apparent potential for fast isotope equilibration in experiments between sulfide and elemental sulfur, non-equilibrium values are observed between the stable sulfur isotope distributions of the same species in many natural systems (Kamyshny and Ferdelman, 2010; Lichtschlag et al., 2013).

Despite the complication of isotope exchange, the use of reduced radiolabeled sulfur compounds has been expedient for showing that sulfide is indeed oxidized to sulfate (Fossing et al., 1992) and for demonstrating the formation of certain intermediates, such as thiosulfate. Experiments in which radioactivity in the thiosulfate pool was trapped by adding a large non-radioactive pool of thiosulfate showed that thiosulfate formed as a key intermediate during sulfide oxidation, in that half of the sulfide oxidized formed thiosulfate, at least transiently (Fossing and Jørgensen, 1990b). Experiments using radiolabeled thiosulfate have furthermore shown that thiosulfate is concurrently oxidized, reduced and disproportionated throughout both oxidized and reduced sediment, the predominant pathway depending on the prevailing redox conditions (Jørgensen, 1990; Fossing and Jørgensen, 1990b).

A variety of experiments and approaches to determine sulfide oxidation have also been conducted without the use of radiotracers. Amendment experiments in which additional oxidants (iron or manganese oxides) were added to sediment incubations (e.g., Burdige and Nealson, 1986; Aller and Rude, 1988; Canfield, 1989; King, 1990) have been used to demonstrate sulfide oxidation and the formation of oxidized products, such as elemental sulfur and sulfate, as discussed above. The quantification of sulfide oxidation intermediates in environmental samples has been used as further evidence for sulfide oxidation, as these intermediates do not typically form during sulfate reduction (e.g., Zopfi et al., 2004). However, the fast turnover of these intermediates may result in low concentrations that belie their significance during sulfide oxidation (Zopfi et al., 2004; Findlay and Kamyshny, 2017). Finally, as discussed in section Stable Sulfur Isotopes, stable sulfur isotopes and modeling of isotopic distributions have been used to gain key insights into sulfide oxidation in marine sediments (e.g.,Dale et al., 2009; Pellerin et al., 2015b).

### Formation of Pyrite

The formation of pyrite (FeS_2_) represents the main burial of sulfur, and thereby of reducing potential, in marine sediments, as pyrite is stable over geological timescales under anoxic conditions (Bottrell and Newton, 2006; Fike et al., 2015). Very generally, pyrite forms from the reaction of sulfide with buried ferric iron minerals, initially forming a mixture of elemental sulfur, polysulfides and ferrous iron minerals. Different overall reactions leading to pyrite formation in marine sediments have been proposed over the years, depending upon the initial reacting iron, and sulfur species. However, it has been argued that despite this potential variety only two reaction mechanisms are important: the reaction between FeS and H_2_S (“H_2_S pathway”; Equation 3) (Rickard and Luther,  1997; Thiel et al., 2019) and the reaction between FeS and polysulfide (“polysulfide pathway”; Equation 4) (Rickard and Luther, 2007).

(3) $$FeS+H_{2}S\to {FeS}_{2}+H_{2}$$

(4) $$FeS+S_{x}^{2-}\to {FeS}_{2}+{S_{x-1}}^{2-}$$

These reaction mechanisms describe the specific step of pyrite formation, rather than the net conversion of iron and sulfide to pyrite (the reaction pathway). The kinetic parameters have been experimentally determined for both reactions (Luther, 1991; Rickard and Luther,  1997). The rate-limiting step is the production and dissolution of FeS and production of reactive sulfur (i.e., polysulfide). Both mechanisms have been confirmed by stable isotope tracer experiments (Butler et al., 2004).

The importance of each mechanism is expected to change based upon environmental parameters such as pH and elemental sulfur concentration. Yücel et al. (2010) modeled the progression of pyrite formation in oxidized turbidite layers in the Black Sea and calculated that, as long as elemental sulfur was present to form polysulfide, S*_x_*^2-^, the polysulfide pathway dominated pyrite formation. Once elemental sulfur was consumed, the H_2_S pathway became more important.

Recently, however, a new reaction mechanism for pyrite formation was proposed, as experimental rates of pyrite formation could not be explained by the traditional model of Rickard (1975). By this new mechanism, surface-complexed Fe(II) reacts with sulfide to form an attached Fe(II)S^2-^ precursor to pyrite. This species then forms FeS_2_ through equilibrium with the aqueous phase (Wan et al., 2017). This reaction mechanism was hypothesized to be particularly important in environments containing high concentrations of ferric iron and low sulfide, for example in surface sediments and deep below the SMT.

In addition to these inorganic experiments and geochemical reactions, microorganisms may play a significant role in pyrite formation in marine sediments (Thiel et al., 2019), although their influence on iron sulfide mineral formation is not straightforward to determine (Picard et al., 2016). For example, elemental sulfur disproportionating bacteria have been shown to increase pyrite formation rates in cultures (Canfield et al., 1998) and possibly also in marine sediments (Zopfi et al., 2008).

### Sulfidization of Organic Matter

The incorporation of sulfide into organic matter may represent a significant sink for sulfide in some marine sediments. Rates may be slower than for pyrite formation so that organic sulfur formation becomes a significant process only once reactive iron is depleted (Sinninghe Damsté et al., 1989). This notion is supported by the isotopic depletion of ^34^S in organic sulfur compounds (Raiswell et al., 1993). However, organic sulfur also forms in sediments in which pyrite formation occurs (Brüchert and Pratt, 1996) and it has particularly been observed to precede or coincide with pyrite formation within the top 10 cm of the sediment (Brüchert, 1998). Organic sulfur formation appears to continue throughout the sediment column and can represent a sink for sulfide once the reactive iron is consumed (Dale et al., 2009).

Relatively few studies have compared the formation of organic and inorganic sulfur in marine sediments. Authigenic organic sulfur has been found to contribute almost as much as pyrite to sedimentary sulfur (Dale et al., 2009). Moreover, the sulfidization of organic matter can affect the reactivity of organic compounds, possibly inhibiting microbial degradation (Eglinton et al., 1994), and can affect the isotopic dynamics of pore water and solid-phase species through isotope exchange (Dale et al., 2009; Raven et al., 2016).

## Stable Sulfur Isotopes

The stable isotope composition of different sulfur species in the seabed provides important information about the current and past biogeochemical sulfur cycle. Sulfur has four naturally occurring stable isotopes with atomic weights (and natural abundances) of 32 (95.02%), 33 (0.75%), 34 (4.21%), and 36 (0.02%) (Coplen and Krouse, 1998). The small mass differences result in isotope fractionation, i.e., differences in the isotopic composition of the product (e.g., sulfide) relative to the reactant (e.g., sulfate). These differences are mostly expressed during microbial processes, such as sulfate reduction, and depend on the environmental conditions under which the microorganisms live. Sulfur isotopes are therefore a useful tool to determine prevailing processes and geochemical conditions in modern and ancient sediments.

The quantification of sulfur isotope fractionation in marine sediments relies on the separation of different sulfur pools. From the pore water, dissolved sulfate and sulfide are typically analyzed. From the solid sediment, iron monosulfides are extracted with HCl (acid volatile sulfides, AVS), while iron disulfide such as pyrite is extracted with reduced chromium in HCl (chromium reducible sulfur, CRS). Elemental sulfur is extracted with organic solvents such as methanol or toluene (Zopfi et al., 2004; Yücel et al., 2010). Different fractions of organic sulfur can be chemically extracted with organic solvents (Oduro et al., 2011). Minor pore water constituents like thiosulfate or sulfite are present in such low concentrations (<1 μM) that measurement of their isotopic composition has not yet been possible in marine sediment.

Once the sulfur pools are separated, the composition of the two most abundant isotopes, ^32^S and ^34^S, is measured by isotope ratio mass spectrometry. The results are typically reported in delta notation, δ^34^S, which shows how many permille the ^34^S/^32^S ratio of a sample deviates from the similar ratio of a standard [the Vienna-Canyon Diablo Troilite international reference scale (V-CDT)]:

(5) δ34S(‰)=([S34/S32]sample[S34/S32]V−CDT−1)×1000

### Sulfur Isotope Fractionation

When sulfur undergoes transformation from an oxidized to a more reduced phase or vice versa, the heavy isotopes generally have a reaction velocity that is slightly slower than the light isotopes (Bigeleisen, 1949). This results in a product that is relatively enriched in the light isotopes relative to the reacting pool. For the reaction A ⇒B, such a unidirectional enrichment is termed kinetic fractionation and is defined as:

(6) $$34_{\alpha_{kin}=\frac{34_{R_{A-B}}}{34_{R_{A}}}}$$

where ^34^α*_kin_* is the kinetic fractionation factor, ^34^R_A-B_ denotes the ^34^S/^32^S ratio of the material converted from the A pool to the B pool, and ^34^R_A_ is the ^34^S/^32^S ratio of the A pool.

Heavy isotopes typically are more stable when strong bonds are formed (such as ^34^S with oxygen) while lighter isotopes are more stable in weaker bonds (such as ^32^S with hydrogen). If both forward and backward reactions occur (A→B and B←A), the two sulfur species may approach thermodynamic equilibrium whereby there is no longer net flow of ^34^S or ^32^S in either direction. In this situation, the isotope ratio between the two species is defined as:

(7) $$34_{\alpha_{equ}=\frac{{34R}_{B}}{{34R}_{A}}}$$

where ^34^α*_equ_* is the equilibrium fractionation factor and ^34^R*_B_* and ^34^R_A_ are the ^34^S/^32^S ratios in pool B and A, respectively. The isotope fractionations by microbial transformation of sulfur species in marine sediments are a combination of kinetic and equilibrium fractionation.

The main process imparting sulfur isotope fractionation in marine sediments is DSR (Figure 4). The net fractionation factor, ^34^*α_net_*, during each of the five steps, from the reactant, r (external SO_4_^2-^) to the product, p (external H_2_S), depends on the degree of reversibility of each reaction step as well as its kinetic and equilibrium isotope fractionation factors (Wing and Halevy, 2014):

(8) $$34_{\alpha_{net}}=(34_{\alpha_{equ}}-34_{\alpha_{kin}})\times f\frac{p}{r}+34_{\alpha_{kin}}$$

where $f\frac{p}{r}$ is the ratio of product formation to reactant formation, i.e., the degree of back-flow during the reaction. The $f\frac{p}{r}$ is connected to the thermodynamic driving force:

(9) $$f_{\frac{p}{r}}=e^{\Delta G_{r}}/RT$$

where ΔG*_r_* is the free energy change (Gibbs energy) associated with the transformation, R is the gas constant and T is the temperature in degrees Kelvin. High substrate concentration and/or low product concentration lead to high thermodynamic driving force and, thus, low net isotope fractionation. The overall fractionation imparted during DSR, ^34^α*_dsr_*, is influenced by the ^34^α*_net_* values of each individual step in the DSR pathway. We refer to this measurable fractionation between sulfide and sulfate during sulfate reduction as ^34^ε, defined as:

(10) $$34_{\varepsilon_{dsr}=(34_{\alpha_{dsr}}-1)*1000}$$

The magnitude of ^34^ε*_dsr_* produced by DSR can theoretically vary between 0 and -70‰. The latter is near the thermodynamic equilibrium value between sulfate and sulfide in typical marine sediment (Tudge and Thode, 1950; Farquhar et al., 2003).

### Sulfate Reduction

From Equations (8) and (9) we can see that the resulting isotope fractionation during sulfate reduction is a function of free energy associated with sulfate reduction, which in turn relates to the extracellular and intracellular environmental conditions under which individual steps in sulfate reduction are taking place (Wing and Halevy, 2014). This relationship provides the possibility to obtain environmental information from sulfur isotope compositions in ancient and modern marine sediments.

**FIGURE 9:**
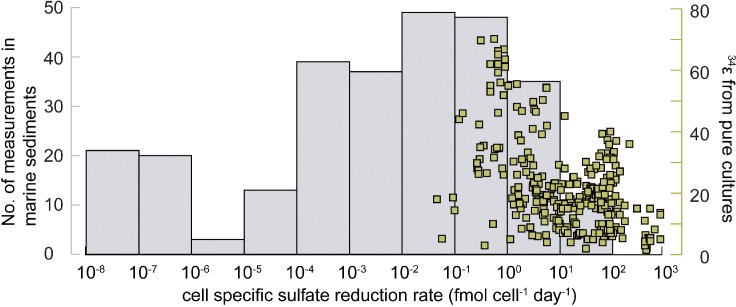
Frequency distribution of mean cell-specific sulfate reduction rates (csSRR). Gray bars (left axis): number of measurements in different csSRR-intervals in marine sediments. Right axis (green squares): Distribution of csSRR in laboratory cultures where also sulfur isotope fractionation factors were determined. Rates compiled for marine sediment are taken from D’Hondt et al. (2002); Leloup et al. (2009), and Beulig et al. (2018), while pure culture data are a non-exhaustive compilation from Kaplan and Rittenberg (1964), Chambers et al. (1975), Detmers et al. (2001), Habicht et al. (2005), Hoek et al. (2006), Johnston et al. (2007), Davidson et al. (2009), Eckert et al. (2011), Sim et al. (2011a,b, 2012), Leavitt et al. (2013), Pellerin et al. (2015a), Antler et al. (2017), Zaarur et al. (2017) and Pellerin et al. (2018b).

Parameters that influence ^34^ε have been primarily studied in laboratory experiments using either pure cultures of SRM or organic-rich sediment. However, most of these experiments were performed at much higher thermodynamic drive, and therefore much higher cell-specific sulfate reduction rates (csSRR), than generally occur in marine sediments (see section Controls on SRM Communities). Figure 9 shows a compilation of 250 published data on mean csSRR from sulfur isotope experiments with pure cultures of SRB and with marine sediments. The mean csSRR of pure cultures range from 0.1 to 1000 fmol SO_4_^2-^ cell^-1^ day^-1^. While there is a large scatter, a negative correlation exists between csSRR and sulfur isotope fractionation in pure cultures. The mean csSRR of marine sediments, in contrast, are generally below 0.1 fmol SO_4_^2-^ cell^-1^ day^-1^ and may drop far below 0.001 fmol SO_4_^2-^ cell^-1^ day^-1^ deep down (Figure 4D, 9; Hoehler and Jørgensen, 2013). Only in the uppermost range of cell-specific rates observed in marine sediments are sulfur isotope fractionation factors available under controlled experimental conditions. This indicates that our understanding of sulfur isotope fractionation in marine sediments is hinged on the assumption that physiological conditions in marine sediment are reflected in pure culture experiments at high rates. That is not exactly the case. It may therefore be inaccurate to use such laboratory-generated ^34^ε values for the interpretation of sulfate reduction under *in situ* conditions in the seabed. The most consistent relationship between an environmental variable and ^34^ε in marine sediments is the dependence on electron donor availability. As mentioned, recalcitrant carbon substrates and continuous low substrate abundances in sediments lead to low csSRR and low Gibbs energy, ΔG*_r_* (Jin and Bethke, 2009), which result in high fractionation, ^34^ε (Harrison and Thode, 1958; Kaplan and Rittenberg, 1964; Chambers et al., 1975; Sim et al., 2011a,b; Leavitt et al., 2013). For example, sulfate reduction experiments with anaerobic oxidation of methane (Deusner et al., 2014) or of H_2_ (Hoek et al., 2006) show that ^34^ε becomes numerically smaller by higher substrate concentration.

### Controls on Isotope Fractionation

The csSRR and ^34^ε are correlated because both are controlled by the fluxes of electron donors and acceptors. As seen above, enhanced substrate supply and higher growth rates tend to reduce ^34^ε. Over many generations living under nutrient replete conditions, natural selection may favor individuals with higher growth rates, which may gradually diminish the overall ^34^ε (Pellerin et al., 2015a). Evolutionary processes may thus over time lead to different ^34^ε under similar environmental conditions, which complicates the interpretation of ^34^ε as an environmental proxy. Similarly, as purifying selection of low-energy adapted communities progresses during their subsurface burial (Starnawski et al., 2017) the overall ^34^ε of DSR could, in principle, gradually change.

In the face of environmental perturbations and the observation that SRB adjust to such perturbations, the relationship between ^34^ε and csSRR is not straightforward but is rather driven by non-steady state in the pathway of DSR. This complication can, on the other hand, yield insights into the cellular machinery and turnover times of intermediates. For example, *Desulfovibrio vulgaris* in batch culture exhibits distinct ^34^ε as a function of growth phase, with low ^34^ε in early exponential phase and high ^34^ε in stationary phase. When transferred to fresh medium, however, the expressed ^34^ε does not immediately reflect the new growth conditions. Rather, a delay is observed in re-adjusting ^34^ε which can even last longer than a generation (Pellerin et al., 2018b).

Sulfate availability also affects ^34^ε. Theoretical work has suggested that ^34^ε can remain large even under very low sulfate concentrations, depending upon the csSRR (Wing and Halevy, 2014). For example, down to a sulfate concentration of 10 μM, *Desulfovibrio vulgaris* is predicted to show little or no variation in ^34^ε due to sulfate limitation when grown at low csSRR typically encountered in the environment (<0.2 fmol SO_4_^2-^ cell^-1^ day^-1^) (Figure 9). In contrast, at high csSRR of ≥5 fmol SO_4_^2-^ cell^-1^ day^-1^ (i.e., at typical laboratory rates of sulfate reduction) a sulfate concentration of 10 μM will strongly diminish ^34^ε because virtually all sulfate which enters the cell is converted to sulfide (a low reversibility in the first step) (Wing and Halevy, 2014; Bradley et al., 2016). These recent findings contradict previous conclusions that low sulfate concentrations consistently diminish ^34^ε (Habicht et al., 2002; Nakagawa et al., 2012; Gomes and Hurtgen, 2013). The earlier studies were done under relatively high organic substrate availability and high csSRR.

**FIGURE 10:**
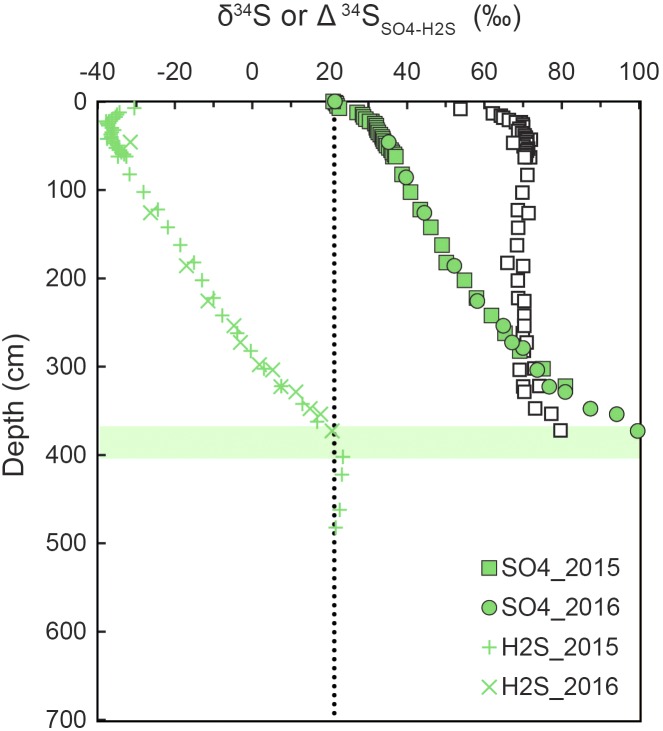
Depth distributions of sulfur isotope compositions (δ^34^S) of sulfide and sulfate at station M24 in Aarhus Bay in two consecutive years. The isotopic difference between pore water sulfate and sulfide is also shown (open squares). A sulfate-methane transition (SMT) is located at 370–400 cm depth. Redrawn from Pellerin et al. (2018a).

Attenuation of ^34^ε because of low sulfate may seldom be expressed under the low csSRR in marine sediments, as the SRM adjust their uptake affinity for sulfate to the ambient concentration (see section Controls on SRM Communities). As an example, ^34^ε was found to be >20‰ in the water column of a low-sulfate lake, even at sulfate concentrations of ≤6 μM (Crowe et al., 2014). Furthermore, pyrite grains highly enriched in ^34^S form at the SMT of sediments from the South China Sea (Lin et al., 2017). This must be the result of a high ^34^ε sustained even at the low sulfate concentrations found in the SMT. Similarly, high ^34^ε at low sulfate occur in the Baltic Sea where large fractionation is still evident near the SMT (Figure 10; Pellerin et al., 2018a).

### Sulfide Oxidation and Disproportionation

Sulfide oxidation can augment or overprint the original isotopic signature of DSR when the two processes occur concurrently. The oxidation of sulfide to intermediate sulfur species or to sulfate appears to produce only small isotope effects (Fry et al., 1986, 1988; Zerkle et al., 2009; Balci et al., 2012). In contrast, the extracellular oxidation of sulfide to an intermediate species (e.g., elemental sulfur) and subsequent disproportionation can induce large fractionations which have been measured up to 30‰ (Canfield and Thamdrup, 1994; Habicht et al., 1998) but could be even higher. In pure cultures grown under excess iron oxides, elemental sulfur disproportionation was found to produce sulfate enriched by up to 18‰ and sulfide depleted -5‰ relative to the starting elemental sulfur, i.e., a Δ^34^S_*SO*4-*H*2*S*_ of 23‰ (Böttcher et al., 2001). This Δ^34^S denotes the difference in isotopic composition between reactant and product sulfur species (δ^34^S_A_ - δ^34^S*_B_*). If disproportionation is quantitatively important it can therefore have a large impact on the δ^34^S systematics in marine sediment. In contrast to sulfide oxidation with Fe(III), oxidation with Mn(IV) results in little or no isotopic fractionation since the sulfide is chemically oxidized all the way to sulfate (Böttcher and Thamdrup, 2001).

Sulfate reduction and sulfur disproportionation operate concurrently in marine sediment and may thereby induce large sulfur isotope fractionations. It is not possible to differentiate the two types of metabolism using only ^34^S/^32^S ratios unless the fractionations appear greater than -70‰, which is the thermodynamic equilibrium for DSR. A tool which has more recently yielded insight into the oxidative sulfur cycle is the measurement of multiple sulfur isotopes (MSI) by which also the minor isotopes, ^33^S (and possibly ^36^S), are considered in addition to ^32^S and ^34^S. The fractionation of ^33^S^/32^S is about half that of ^34^S^/32^S, owing to the mass differences between the sulfur isotopes. Yet, the difference, which can be very accurately determined, is not exactly half, and this slight deviation from half holds important information about the current or past sulfur transformations.

By a transformation from sulfur compound A to B, e.g., from sulfate to sulfide, the ratio of fractionation factors for ^33^S/^32^S and ^34^S/^32^S can be described by their natural logarithm:

(11) $$\theta =\frac{\text{In }\left(33_{\alpha_{B-A}}\right)}{\text{In }\left(34_{\alpha_{B-A}}\right)}$$

Kinetic fractionation by unidirectional processes generates θ values varying between 0.500 and 0.510 whereas equilibrium fractionation produces a more constrained θ value of 0.515 (0.514–0.516) (Farquhar et al., 2003, and references therein). The measurement of MSI can thereby provide information about the reversibility of microbial sulfur transformations. The MSI also open the possibility to discriminate sediment processes in metabolic networks, including concurrent sulfate reduction, sulfide oxidation and disproportionation (Farquhar et al., 2003; Johnston et al., 2005).

MSI data are generally reported as Δ^33^S values. In contrast to Δ^34^S mentioned above, Δ^33^S is used to denote the difference for a single sulfur species between its actual isotopic composition, δ^33^S, and its theoretical isotopic composition, assuming a relationship by thermodynamic equilibrium fractionation between ^33^S and ^34^S of 0.515:

(12) $$\Delta^{33}S=\delta^{33}S-1000\times \left({\left(1+\frac{\delta^{34}S}{1000}\right)}^{0515}-1\right)$$

MSI have been measured in a variety of modern marine sediments, for example the Baltic Sea (Strauss et al., 2012), Mangrove Lake (Pellerin et al., 2015b), Alfonso Basin (Masterson et al., 2018), and South China Sea (Lin et al., 2017, 2018). In a sapropel sediment of Mangrove Lake, Bermuda, the Δ^33^S of pore water sulfate was inconsistent with only sulfate reduction. By combining the MSI signatures of sulfate reduction, disproportionation and sulfide oxidation measured in pure cultures, it was calculated that 50–80% of the sulfate reduced to sulfide returned to sulfate via reoxidation and disproportionation (Pellerin et al., 2015b). Such a distinction between processes requires that the reactions occur far from equilibrium, i.e., at low ^34^ε of 0–40‰, which is not typical of most marine sediments (Johnston et al., 2005; Pellerin et al., 2015b).

Although MSI provide an independent tool to track microbial sulfur cycling in sediments, it is important to consider the potential complexity of processes in the interpretation of Δ^33^S data. For example, based on a pure culture metabolic model, low Δ^33^S was interpreted as a combined result of sulfate reduction, sulfide oxidation and disproportionation in a surface sediment of the South China Sea (Lin et al., 2017). However, other processes could also produce the same MSI signature which would masquerade as disproportionation. For example, another explanation could be mixing between authigenic pyrite formed *in situ* by sulfate reduction (low δ^34^S and high Δ^33^S) and metal sulfide with a δ^34^S and Δ^33^S of 0‰ from an external, igneous origin deposited with the sediment.

### Isotope Dynamics in Marine Sediments

The isotopic composition of sulfate, sulfide and other sulfur species in a sediment horizon is a result of (a) the ongoing processes such as sulfate reduction, sulfide oxidation and disproportionation, (b) past processes in the same sediment, which at some earlier stage precipitated sulfide as pyrite or formed elemental sulfur and organic sulfur, and (c) communication with the isotopic signals and fractionation processes in other sediment horizons through diffusion of sulfate and sulfide.

A key to understand this complexity is the openness of marine sediments to solute exchange through the pore fluid (Goldhaber and Kaplan, 1980). It is clear that the surface sediment is in open exchange with the overlying seawater since there is only little sulfate depletion despite very active sulfate reduction (Figure 4). Sulfide is also readily oxidized to sulfate in this zone where the benthic macrofauna actively pumps down oxygen and by sediment reworking mixes down oxidized manganese and iron minerals. A part of the sulfide precipitates here as amorphous iron sulfide and pyrite. The difference between the isotopic compositions of pore water sulfate and pyrite may therefore be a good estimator of ^34^ε at the sediment surface (Goldhaber and Kaplan, 1980).

It is less intuitive that in deeper sediments the isotopes of its pore water solutes are in open exchange with the sediment above and below. The exchange takes place through slow molecular diffusion along the gradients of sulfate and sulfide. Interestingly, the gradual increase in δ^34^S of both sulfate and sulfide with depth (Figure 10) means that the relative diffusion gradients of ^32^S and ^34^S differ from their relative concentrations in the bulk sulfate and sulfide. Since sulfate becomes relatively enriched in ^34^S with depth, the ^34^S concentration does not drop off as steeply as the ^32^S concentration, i.e., ^32^S has a relatively steeper diffusion gradient than does ^34^S. Sulfate diffusing downwards through a given sediment horizon therefore has a ^32^S-enriched isotopic composition relative to the sulfate present at that horizon. The opposite is the case for sulfide diffusing upwards, which is enriched in ^34^S. As a result, the isotopic composition of sulfate diffusing down approaches the isotopic composition of sulfide diffusing up, thereby maintaining isotope mass balance between influx of sulfate, total sulfur burial, and outflux of sulfide (Jørgensen, 1979). This effect on the diffusing sulfate and sulfide is exclusively due to the difference in relative concentration gradients of ^32^S and ^34^S and is not due to a difference in the diffusion coefficients of the two isotopes (Wortmann and Chernyavsky, 2011). The same principle applies for the ^33^S isotope and thus for Δ^33^S (e.g., Masterson et al., 2018).

As a conclusion, the increase in δ^34^S of sulfate with depth cannot be used to calculate sulfate reduction according to a closed system model as one might do, for instance, in a bottled pure culture (Pellerin et al., 2015a). If the open diffusion exchange were neglected, it would lead to an underestimation of ^34^ε associated with microbial sulfate reduction. As one type of evidence for openness of the sulfur cycle, mass balance shows that the amount of sulfur trapped in sediments as pyrite usually far exceeds the sulfate that was initially trapped in the pore water during deposition of the sediment (Goldhaber and Kaplan, 1980).

### Solid Phase Sulfur Formation

Sulfide precipitated in sediment as pyrite is stable on geological timescales, which makes the preserved sulfur isotope signatures useful as biological markers and proxies for the early development of ocean chemistry and oxygenation of Earth’s atmosphere. The great quantities of ^34^S-depleted sulfur buried as sedimentary sulfides are also the reason why seawater sulfate is enriched in ^34^S (δ^34^S = +21‰) relative to the bulk solar system value (δ^34^S = 0‰).

The precipitation of sulfide minerals is associated with only negligible fractionations (Price and Shieh, 1979; Wilkin and Barnes, 1996; Böttcher et al., 1998), yet the pathway of pyrite formation (section Formation of Pyrite) affects its preserved sulfur isotope signature. By the H_2_S pathway of pyrite formation (Equation 2) the pyrite has the same δ^34^S as H_2_S, while by the polysulfide pathway (Equation 3) it has a mixed δ^34^S from the FeS precursor and H_2_S and elemental sulfur (Butler et al., 2004). As the bulk pyrite does not exchange isotopes with ambient reduced sulfur (cf. section Formation of Pyrite) (Raven et al., 2016), the δ^34^S of buried pyrite reflects the δ^34^S of sulfide and of elemental sulfur (depending on the pathway) where it was first formed. Pyrite in subsurface sediments is therefore generally ^32^S-enriched relative to the ambient pore water sulfide. As the sediment is buried deeper, the additional pyrite formed becomes progressively enriched in ^34^S.

In many coastal sediments, such as the Baltic Sea and the Black Sea, the modern marine sediments that were deposited since the last ice age overlay late-glacial clay rich in iron but poor in organic matter. This deep clay today functions as a sink for H_2_S which diffuses downwards to reach a sulfidization front, resulting in intensive formation of elemental sulfur and iron sulfides (Böttcher and Lepland, 2000; Jørgensen et al., 2004; Neretin et al., 2004; Holmkvist et al., 2014). The isotopic composition of the pyrite formed at this front is similar to that of the sulfide at the SMT (cf. Figure 10). The isotope data thus provide information on where the sulfide was formed, how it was transported in the sediment, and where it was trapped as iron sulfide minerals. Such observations are important for the correct interpretation of ocean chemistry from δ^34^S in sulfide minerals from the rock record.

## Synthesis and Future Directions

Our understanding of the biogeochemical sulfur cycle of marine sediments has developed in many directions in recent years. New techniques and approaches have been introduced based on DNA and RNA analyses, single-cell studies, high-resolution chemical and isotopic analyses, new experimental methods, and mathematical models. These studies have expanded our functional and quantitative understanding of the sulfur cycle and brought exciting discoveries. In the following, we will briefly outline some examples of the progress, indicate some remaining open questions, and suggest some important directions of future research.

### Microbiology

An overarching breakthrough in microbial ecology has been the development of high-throughput sequencing techniques for DNA and RNA and the broad range of molecular approaches based on the rapidly growing database of sequence information. Techniques for quantitative PCR, fluorescence *in situ* hybridization, single-cell genome sequencing etc. are behind much of we now know about genes, cells and communities of microorganisms engaged in the marine sulfur cycle (e.g., Wasmund et al., 2017). The great capacity for metagenomic sequencing to identify metagenome-assembled genomes has led to the discovery of many uncultured lineages of bacteria and archaea, e.g., with the capacity for sulfate reduction (Anantharaman et al., 2018). We are still far from understanding the importance of such a large microbial diversity for the function of the marine sulfur cycle.

Concurrent quantification of physiologically defined microorganisms, e.g., of sulfate reducers, and measurements of their sulfate reduction rate has opened the possibility to calculate mean metabolic rates per cell for an entire community (e.g., Hoehler and Jørgensen, 2013). It is a question by such mean calculations whether all cells are (equally) active and whether the calculated low metabolic rates enable growth and cell division. Experimental demonstration of the assimilation of isotope-labeled substrates into lipids or into single cells has indicated that many or most microbial cells, even in deep sub-surface sediment, are metabolically active (e.g., Trembath-Reichert et al., 2017). Similar approaches can be used to study the growth and turnover of cells that are specifically active in the sulfur cycle. Altogether, important discoveries in the microbial sulfur cycle will come from a combination of different approaches from biochemistry, physiology, cultivation, and whole sediment experiments, all of which are needed to make further progress.

### Sulfate Reduction

Among the advances in understanding sulfate reduction was the recognition that the initial hydrolytic breakdown of complex organic molecules is rate-limiting by organic matter degradation (e.g., Arnosti, 2004) and that degradation rates seem unaffected by the type of terminal mineralization step, i.e., sulfate reduction or methanogenesis (Beulig et al., 2018). The terminal step is controlled by the production rate of fermentation products, specifically of H_2_ and acetate that may be utilized by either sulfate reducers or methanogens (e.g., Capone and Kiene, 1988). These products have turnover times of hours to years, yet they are generally maintained by the consumers at a low nM (H_2_) or μM (volatile fatty acids, VFAs) concentration. The H_2_ and the VFA concentrations appear to be controlled by the terminal consumers (Hoehler et al., 1998; Glombitza et al., 2015), yet the control mechanisms under low-energy conditions in subsurface sediments are not well understood (Jin and Bethke, 2009). Continuous culture experiments at low dilution rates that approach environmental conditions may help determine the metabolic control on VFA uptake and also on cryptic sulfate reduction below the SMT (Pellerin et al., 2018a).

Furthermore, new approaches and ideas are needed to study microbial adaptations to minimum power and substrate availability such as those found in the seafloor. Microbial life in the subsurface seabed is characterized by extremely low metabolic rates and long generation times (Lomstein et al., 2012). Ideally, the rate of energy metabolism and growth should be studied both at the community and the single-cell level in order to understand the regulation of community size and the spectrum of cellular metabolic rates. Sulfate reducing bacteria are probably better suited than sulfide oxidizers for such studies because they can be identified and quantified by their diagnostic genes, and their sulfate respiration can be measured by the sensitive ^35^S-method (Müller et al., 2014; Røy et al., 2014). Similar molecular markers universal for sulfide oxidizing microorganisms are currently not known (Wasmund et al., 2017), and sulfide oxidation is difficult to measure accurately in sediments (Fossing and Jørgensen, 1990b).

A remaining unresolved question relates to the sulfate and methane profiles in marine sediments, which indicate that anaerobic oxidation of methane (AOM) is an important sink for sulfate. Yet, the methane flux corresponds to only 3–4% of the global organic carbon flux to the seafloor (Egger et al., 2018). The sulfate flux into the SMT generally exceeds the methane flux (Egger et al., 2018) because the sulfate reduction is fed both by AOM and by the degradation of organic matter (Beulig et al., 2019). A poorly constrained, but potentially large fraction of the entire methane production may take place within the SMT as a cryptic methane cycle that is not distinguishable from organoclastic sulfate reduction (cf. Beulig et al., 2019). This illustrates the need for accurate, high-resolution rate measurements of these processes to accompany reaction-transport modeling and for a better microbiological understanding of the combined methanogenesis and AOM.

Finally, despite recent progress, it is still unknown what causes the discrepancy between measured and modeled SRR. Sulfate reduction in marine sediments is strongly focused, (a) toward the ocean margins with high depositional rates (Egger et al., 2018), and (b) toward the surface zone with complex bioirrigation, sediment reworking and sulfide reoxidation (Dale et al., 2019). This complexity causes a general discrepancy between modeled net rates and ^35^S-measured gross rates of sulfate reduction, both on a local and a global scale (e.g., Canfield et al., 2005; Jørgensen and Parkes, 2010; Bowles et al., 2014). Further research on sediment reworking and irrigation by benthic fauna and on sulfide recycling is needed to reconcile SRR determined by the two approaches (Dale et al., 2019). Furthermore, enzymatic back-reaction during microbial sulfate reduction could lead to an overestimation of rates determined by the ^35^S technique. The extent of this back-reaction under different sediment conditions is important to understand because it not only affects ^35^S experiments but also, as discussed below, controls the isotope fractionation during sulfate reduction (Wing and Halevy, 2014).

### Sulfide Oxidation

Research has demonstrated that sulfide oxidation occurs throughout the sediment column but is most active in the surface zone where bioirrigation and sediment reworking transports oxidants down into contact with free sulfide or iron sulfides. Recently, it was shown that diverse heterotrophic and autotrophic sulfide oxidizers in this zone are responsible for a dark CO_2_ fixation that contributes significantly to the organic carbon budget (Boschker et al., 2014). Furthermore, the recently discovered “cable bacteria,” which are related to sulfate reducers, form several-cm long chains and can conduct an electron current, thereby oxidizing sulfide over “long distance” (Pfeffer et al., 2012; Nielsen and Risgaard-Petersen, 2015).

The pathway from sulfide to sulfate, the coupling between sulfide oxidation and iron reduction, and the quantitative role of these processes in different types of sediment are not well understood. The pathways of sulfide oxidation in the underlying, anoxic sediment are also complex and involve both abiotic reactions and microbial metabolism. Research is needed with the objective to distinguish microbiology and geochemistry by sub-seafloor sulfide oxidation. Among the challenges is that the *in situ* process of sulfide oxidation is very sensitive to experimental manipulation of the sediment and that isotope exchange reactions blur the pathways and rates of sulfur transformation if studied by stable isotopes or by ^35^S-radiotracer (Fossing et al., 1992). New experimental approaches are needed to solve these problems and to support the interpretation of geochemical sulfur data from modern marine sediments or from sedimentary rocks.

It has been shown that the major inorganic intermediates of sulfide oxidation (elemental sulfur, polysulfides, thiosulfate, and sulfite) can be oxidized, reduced or disproportionated, thereby forming a complex network of pathways that also involve iron-sulfur minerals and other sulfur species (Jørgensen and Nelson, 2004). However, although several of these potential processes, and partly also the microbial physiology behind them, have been identified their quantitative role and regulation are not well understood. For example, elemental sulfur is a main product of the chemical sulfide oxidation by Fe(III) (Wan et al., 2014), but it is not known to which extent microorganisms are involved and can potentially affect the process in marine sediments. Experiments to demonstrate specific processes in the pathway of sulfide oxidation have often been done successfully by amending the sediment with substrate for that process, e.g., for elemental sulfur disproportionation (Canfield and Thamdrup, 1996). This, however, potentially changes its rate and balance relative to other pathways, such as the turnover of thiosulfate (Jørgensen, 1990), and it is therefore important to design future experiments with minimal disturbance of the sediment.

Most processes of sulfide oxidation in marine sediments involve microorganisms, sometimes in unexpected ways. A recent example is pyrite formation by the “H_2_S pathway” (section Formation of Pyrite, Equation 3) for which enrichment experiments showed that the process was highly stimulated by H_2_-consuming archaea (Thiel et al., 2019). A growing number of anaerobic microbial processes is found to involve DIET through specialized structures on the cell surface (e.g., Summers et al., 2010). In some cases, the electron transfer is not direct but takes place via microscopic, conductive particles in the sediment, such as pyrite, magnetite or black carbon (Rotaru et al., 2018). We expect that such microbe-mineral interactions will be an important and rewarding research object in the future, for example to understand sulfide oxidation in anoxic sediments.

### Sulfur Isotopes

Recent research has refined our understanding of the intracellular processes that lead to large variations in sulfur isotope fractionation by sulfate reducing microbes (Wing and Halevy, 2014; Leavitt et al., 2015; Sim et al., 2017, 2019). However, most culture studies and many sediment experiments are performed under relatively high substrate levels and correspondingly high energetic drive (e.g., Sim et al., 2011b). Subsurface marine sediments, in contrast, are characterized by organic carbon limitation, low ΔG*_r_*, and low csSRR (LaRowe and Amend, 2015). Back-reaction during sulfate reduction is therefore relatively high in sediments where sulfur isotope fractionation may approach the equilibrium fractionation of ^34^ε = ca 70 (Farquhar et al., 2003). Furthermore, the intracellular equilibration between the sulfur oxy-anions (Ames and Willard, 1951) may be important in the resulting ^34^ε (e.g., Leavitt et al., 2015; Sim et al., 2019). Elucidating the intracellular processes which result in the net ^34^ε will lead to a better understanding of the sulfur cycle in sedimentary environments. Thus far, the intracellular “black box” has made it challenging to infer variations in environmental conditions based on the modest variability of ^34^ε in the seabed (Masterson et al., 2018). Further experimental work, including chemostat cultures under low cell-specific SRR and under variable substrate concentrations, should be combined with theoretical work that takes microbial physiology and thermodynamics into account. Earlier interpretations of the rock record and of paleoceanographic conditions may thereby change in the light of new advances in our understanding of factors controlling sulfur isotope fractionation.

Experimental studies of the rates and pathways of sulfide oxidation have been done with ^35^S-radiotracers rather than with ^34^S-labeled sulfur species. The two have not been combined. Yet, parallel experiments with ^35^S or ^34^S amended sediment could help understand important aspects of the sulfur cycle, such as (a) the isotope exchange between sulfide, elemental sulfur and iron sulfide or (b) the distinction between back-reaction and sulfide re-oxidation during sulfate reduction.

### Sulfur Cycling in the Anthropocene

As discussed in section Sulfate Reduction Rates (SRR), sulfate reduction in the seabed is strongly focused toward near-surface sediments with high depositional rates along the ocean margins. The benthic marine sulfur cycle is therefore sensitive to anthropogenic influence, such as ocean warming and increased nutrient loading of coastal seas. This stimulates photosynthetic productivity and results in enhanced export of organic matter to the seafloor, often combined with low oxygen concentration in the bottom water (Rabalais et al., 2014; Breitburg et al., 2018). The biogeochemical zonation is thereby compressed toward the sediment surface, and the balance of organic matter mineralization is shifted from oxic and suboxic processes toward sulfate reduction and methanogenesis (Middelburg and Levin, 2009).

Whereas these trends are documented by many examples, their future quantitative consequences remain difficult to predict. Eutrophication of coastal waters enhances the importance of sulfate reduction in regulating the mineralization of deposited organic matter (e.g., Jørgensen, 1980; Sampou and Oviatt, 1991). Sulfate reduction thereby also gains a key role in regulating the fraction of organic matter that is buried. The further development of this change in the marine carbon cycle is uncertain as it has happened only within the past century and has affected only the top few centimeters to decimeters of the seabed. A targeted survey of this effect with methods that can resolve the processes in near-surface sediments is needed to understand its local and global significance.

Enhanced sulfate reduction causes enhanced sulfide production in the near-surface sediment. This may partly exhaust the metal oxides, which are otherwise maintained in an oxidized state by irrigation and sediment reworking by benthic fauna (e.g., van de Velde and Meysman, 2016). The effect on the pathways and rates of sulfide oxidation are incompletely understood, but in general the changes in carbon, sulfur and iron biogeochemistry reduce the buffer capacity of sediments to retain sulfide (Kristiansen et al., 2002). Cable bacteria and large vacuolated sulfur bacteria thereby gain importance as ultimate barriers against seasonal sulfide release, as long as some oxygen or nitrate is still available in the bottom water (Seitaj et al., 2015). A quantitative and functional understanding of these processes in eutrophic coastal waters may help to predict the sediment buffer capacity against sulfide release, which could potentially cause fish kills and other adverse environmental effects (Diaz and Rosenberg, 2008).

## Author Contributions

The authors contributed equally to the planning and writing of this review.

## Conflict of Interest Statement

The authors declare that the research was conducted in the absence of any commercial or financial relationships that could be construed as a potential conflict of interest.
